# Deletion of *Gba* in neurons, but not microglia, causes neurodegeneration in a Gaucher mouse model

**DOI:** 10.1172/jci.insight.179126

**Published:** 2024-11-08

**Authors:** Hannah B.D. Duffy, Colleen Byrnes, Hongling Zhu, Galina Tuymetova, Y. Terry Lee, Frances M. Platt, Richard L. Proia

**Affiliations:** 1 Genetics of Development and Disease Section, Genetics and Biochemistry Branch, National Institute of Diabetes and Digestive and Kidney Diseases, NIH, Bethesda, Maryland, USA.; 2 Department of Pharmacology, University of Oxford, Oxford, United Kingdom.

**Keywords:** Genetics, Neuroscience, Genetic diseases, Lysosomes, Neurodegeneration

## Abstract

Gaucher disease, the most prevalent lysosomal storage disease, is caused by homozygous mutations at the *GBA* gene, which is responsible for encoding the enzyme glucocerebrosidase. Neuronopathic Gaucher disease is associated with microgliosis, astrogliosis, and neurodegeneration. However, the role that microglia, astrocytes, and neurons play in the disease remains to be determined. In the current study, we developed inducible, cell-type-specific *Gba*-KO mice to better understand the individual impacts of *Gba* deficiencies on microglia and neurons. *Gba* was conditionally knocked out either exclusively in microglia or neurons or throughout the body. These mouse models were developed using a tamoxifen-inducible Cre system, with tamoxifen administration commencing at weaning. Microglia-specific *Gba*-KO mice showed no signs of disease. However, the neuron-specific *Gba* KO resulted in a shortened lifespan, severe weight loss, and ataxia. These mice also had significant neurodegeneration, microgliosis, and astrogliosis accompanied by the accumulation of glucosylceramide and glucosylsphingosine, recapitulating Gaucher disease–like symptoms. These surprising findings reveal that, unlike the neuron-specific *Gba* deficiency, microglia-specific *Gba* deficiency alone does not induce disease. The neuronal Gaucher disease mouse model, with a median survival of 16 weeks, may be useful for future studies of pathogenesis and the evaluation of therapies.

## Abstract 2

Duffy et al. investigated how neurons and microglia contribute to Gaucher disease and found that microglia have little role on disease onset.

## Introduction

Gaucher disease (GD) has a prevalence of 1 in 800 among Ashkenazi Jews and 1 in 40,000–50,000 in the general population (1). It is caused by mutations in the *GBA* gene, which codes for the enzyme glucocerebrosidase (GCase). GCase is involved in glycosphingolipid catabolism, cleaving glucose from glucosylceramide (GlcCer) to produce ceramide (Cer). GlcCer can also be converted into glucosylsphingosine (GlcSph) via the enzyme acid ceramidase (2). In GD, insufficient quantities of GCase are expressed, leading to an accumulation of GlcCer and GlcSph (3–5).

GD is characterized by hepatosplenomegaly, cytopenia, anemia, and bone disease (1, 6, 7). The least severe presentation of GD, type 1 GD, is usually not associated with neurological symptoms, and individuals with this disease type have the longest lifespan. However, GD types 2 and 3 are neuronopathic. Type 2 GD, the most severe disease type, can result in hepatosplenomegaly, opisthotonos, apnea, bulbar signs, oculomotor paralysis, and myoclonic epilepsy, but bone disease is absent (1, 7). Symptom onset tends to occur at 3–6 months of age, and median lifespan is 9 months (1, 7). In type 3 GD, symptoms typically present by 20 years of age, with half of patients presenting before the age of 2 (7). Those with type 3 GD may live to early adulthood (1). Symptoms vary considerably and may include hepatosplenomegaly, cytopenia, anemia, bone disease, ophthalmoplegia, kyphosis, hydrocephaly, cerebellar ataxia, myoclonic epilepsy, and dementia (7).

The first attempt to model GD in mice resulted in lethality shortly after birth due to rapid water loss across the skin barrier (8). In mice, the conversion of GlcCer to Cer is vital for epidermal maturation (9). The *Gba-*null allele prevented this conversion, impeding the development of the epidermal barrier. Insertions of point mutations linked to GD types 2 and 3 (RecNcil and L444P) had similarly short lifespans due to a similar skin defect (10). Other point mutations (V394L, D409H, and D409V) led to longer lifespans but no neurological substrate accumulation or abnormalities (11). Since these initial attempts, researchers have developed conditional *Gba-*KO models in order to avoid affecting the epidermis. The current conditional KO models for neuronopathic GD, however, do not survive for more than 4 weeks (12). While these neuronopathic models have proven useful, their short lifespans are limiting. More recently, double mutant mice with saposin C deficiencies (D409H:C*) have been proposed as a model for neuronopathic GD (13). Saposin C is an activator protein needed for presenting GlcCer to GCase, and when mutated, it can cause a rare form of GD (14). These mice experience neurological abnormalities in addition to accumulating GlcCer and GlcSph in the brain (13). While their long lifespans (~13 months) may prove useful for some experiments, the saposin C deficiency may not recapitulate the mechanisms underlying common neuronopathic GD mutations. Another recent study made use of a doxycycline-inducible *Gba* transgene to modulate levels of GCase as a model for type 3 GD (15). These mice exhibit skeletal abnormalities, motor deficits, microgliosis, and neurodegeneration, particularly within the cerebellum, and can live up to 10 months. There remains a need for additional genetic models with longer lifespans for extended experimentation.

Gaucher cells, engorged macrophages containing an excess of sphingolipids, are a hallmark of GD (3, 16–18). These enlarged macrophages are thought to cause the hepatosplenomegaly seen in the disease. Enzyme replacement therapy (ERT) targets these macrophages using recombinant GCase with mannose-terminated oligosaccharides. This therapy is successful in treating type 1 GD but not in treating neurological symptoms owing to its inability to cross the blood-brain barrier (19, 20). Efforts to deliver therapies across the blood-brain barrier are ongoing and include substrate reduction therapy, ERT, and gene therapy (21–25).

In order to develop targeted treatment strategies for neuronopathic GD, there remains a need to better understand the neurological mechanisms of the disease. This need has become especially pressing since the discovery that *GBA* mutations are also linked to a more common neurodegenerative disease, Parkinson’s disease (PD). Approximately 7%–20% of patients with PD have heterozygous *GBA* mutations (26). As such, understanding the neurological mechanisms behind GCase deficiencies in the homozygous and heterozygous state is of paramount importance.

Patients with neuronopathic GD exhibit microglial nodules, astrogliosis, and neuronal loss (3, 17, 27, 28). Additionally, brains of both individuals with PD and pharmacologically induced GCase-deficient (conduritol B epoxide–treated [CBE-treated]) mice exhibit lipid storage in both neurons and microglia but not in astrocytes of the substantia nigra (29). As such, both microglia and neurons are implicated in disease progression. Furthermore, as microglia are specialized macrophages that play a similar role, they are a candidate to feature prominently in the disease. However, it is not fully understood to what extent microglia do in fact contribute to disease onset.

A conditional *Gba*-KO mouse targeted to microglia with a CX_3_Cr1 promoter has been reported to result in GlcCer and GlcSph accumulation and motor deficits within a year (30). However, this promoter is also expressed in some macrophages and other leukocytes, making it difficult to differentiate between resident microglia and infiltrating macrophages (31). Additionally, the CX_3_Cr1 promoter has been reported to have leaky expression in neurons, further confounding these results (32). There remains a need for a microglia-specific *Gba*-KO (*Gba*-mKO) mouse to conclusively determine whether this phenotype is due to the microglial deficiency alone.

Two conditional mice have been developed by Enquist et al. (12) to excise *Gba* in neurons. The K14-lnl/lnl mouse results in a *Gba* deletion in all tissues except the skin, while the Nestin-Cre mouse specifically targets neurons and macroglia but not microglia (12). These mice accumulate GlcCer, exhibit neurodegeneration and microgliosis, and develop seizures. While these models effectively target the neuron, a conditional KO exclusive to neurons has not yet been developed. Thus, to what extent this phenotype is due to neurons alone remains elusive.

**Figure 1:**
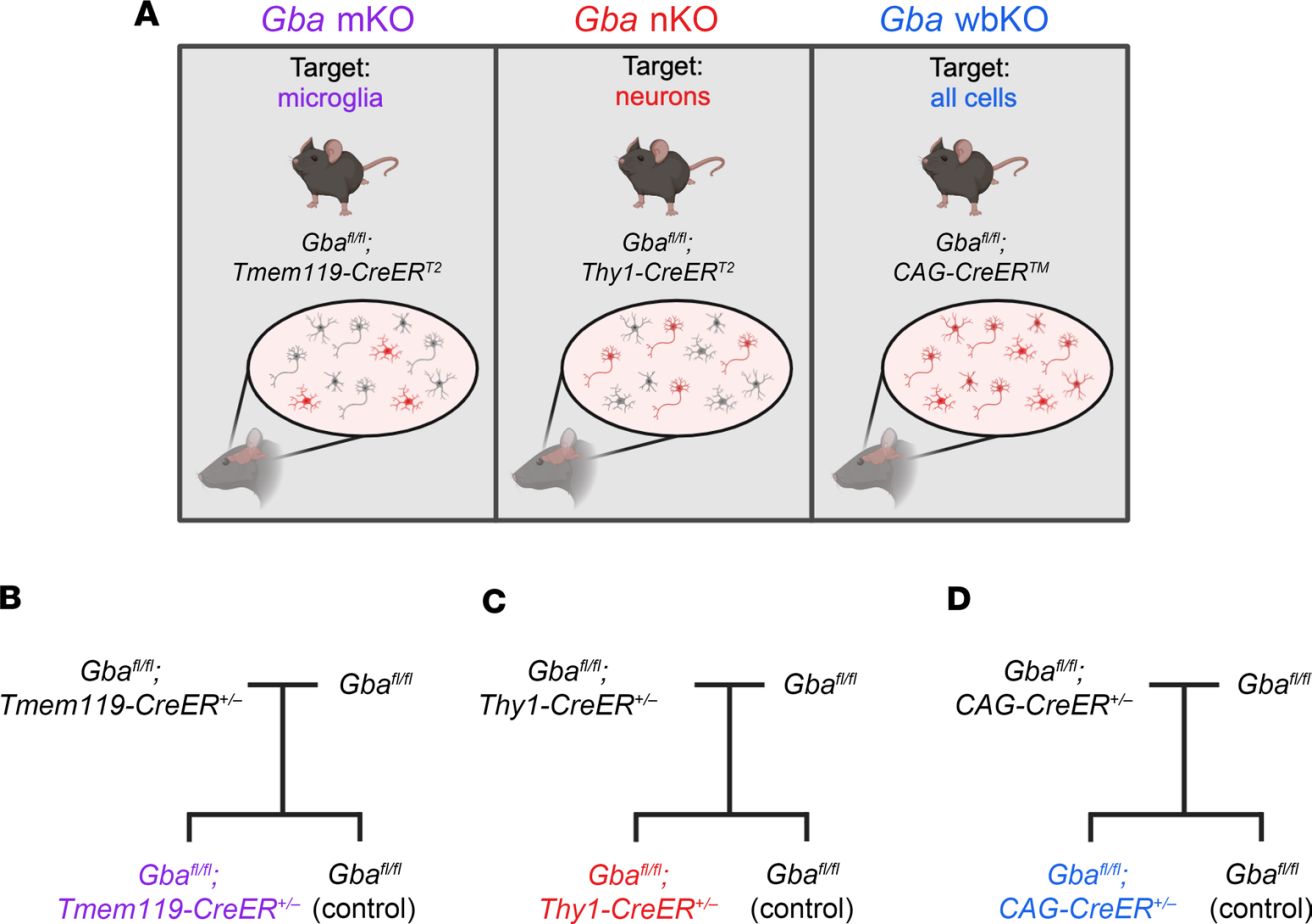
Schematic depicting the experimental design. (**A**) Microglia-specific, neuron-specific, and ubiquitous *Gba*-deficient mice were developed using the *Tmem119*, *Thy1*, and *CAG* promoters, respectively. (**B**–**D**) Schematics illustrating the breeding scheme used to generate *Gba*-mKO (**B**), *Gba*-nKO (**C**), and *Gba*-wbKO (**D**) mice with littermate controls. This figure was created with Biorender.com.

The current study aims to explore how a GCase deficiency within microglia and neurons individually affects disease progression by developing what we believe to be the first *Gba*-KO mouse models exclusive to these cell types (Figure 1). Using cell-type-specific tamoxifen-inducible Cre lines, *Gba* was knocked out either only in microglia or only in neurons. Additionally, to the best of our knowledge, this study provides the first characterization of two mouse models of neuronopathic GD with substantially longer lifespans than most models currently used in the field, providing an option for future long-term studies. Our findings contribute to the evaluation of which cells and mechanisms should be targeted in future therapeutic strategies.

## Results

### The Gba KO is cell-type specific.

In order to verify the specificity of the 3 mouse models, each Cre line (*Tmem119-CreER^T2^*, *Thy1-CreER^T2^,-EYF*P, *CAG-CreER^TM^*) was crossed to a tdTomato reporter [*Ai9*(*RCL-tdT)*] mouse, which contained a floxed stop codon preceding the tdTomato sequence. Cell-specific Cre lines would thus express tdTomato only in cells in which Cre was expressed. These mice were administered a tamoxifen-containing diet at 3 weeks of age and were euthanized at 6 weeks of age.

**Figure 2:**
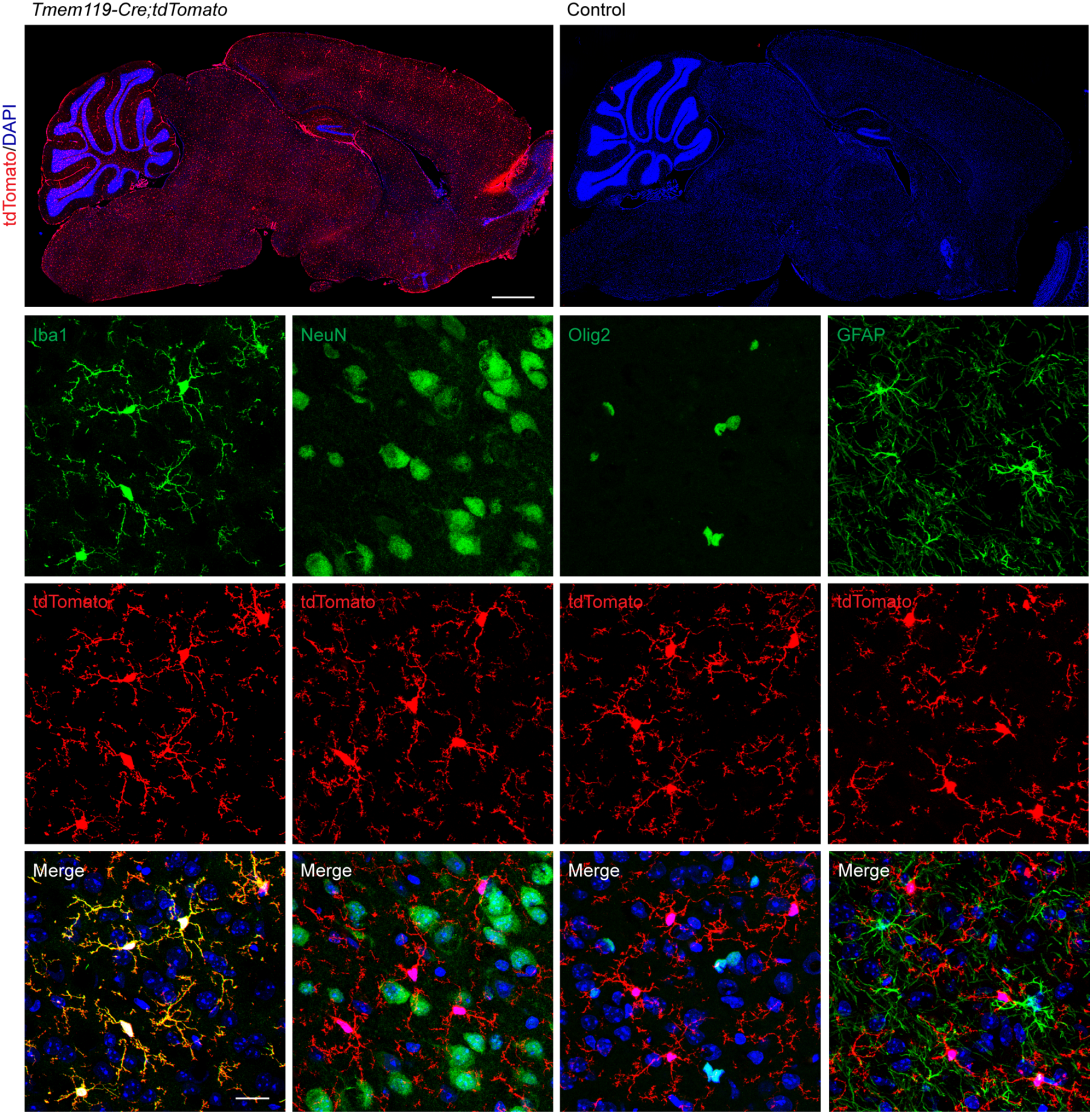
Verification of the cell specificity of the microglia-specific *Gba*-KO mouse. *Tmem119-Cre* mice were crossed with tdTomato reporter mice. *Tmem119-Cre* drives the specific expression of tdTomato in microglia (Iba1 labeled) but not in neurons (NeuN labeled), oligodendrocytes (Olig2 labeled), or astrocytes (GFAP labeled). All images (other than tile scans) are from the cortex. Scale bars: 1 mm (tile scan); 20 μm (bottom 3 rows). *n* = 3.

**Figure 3:**
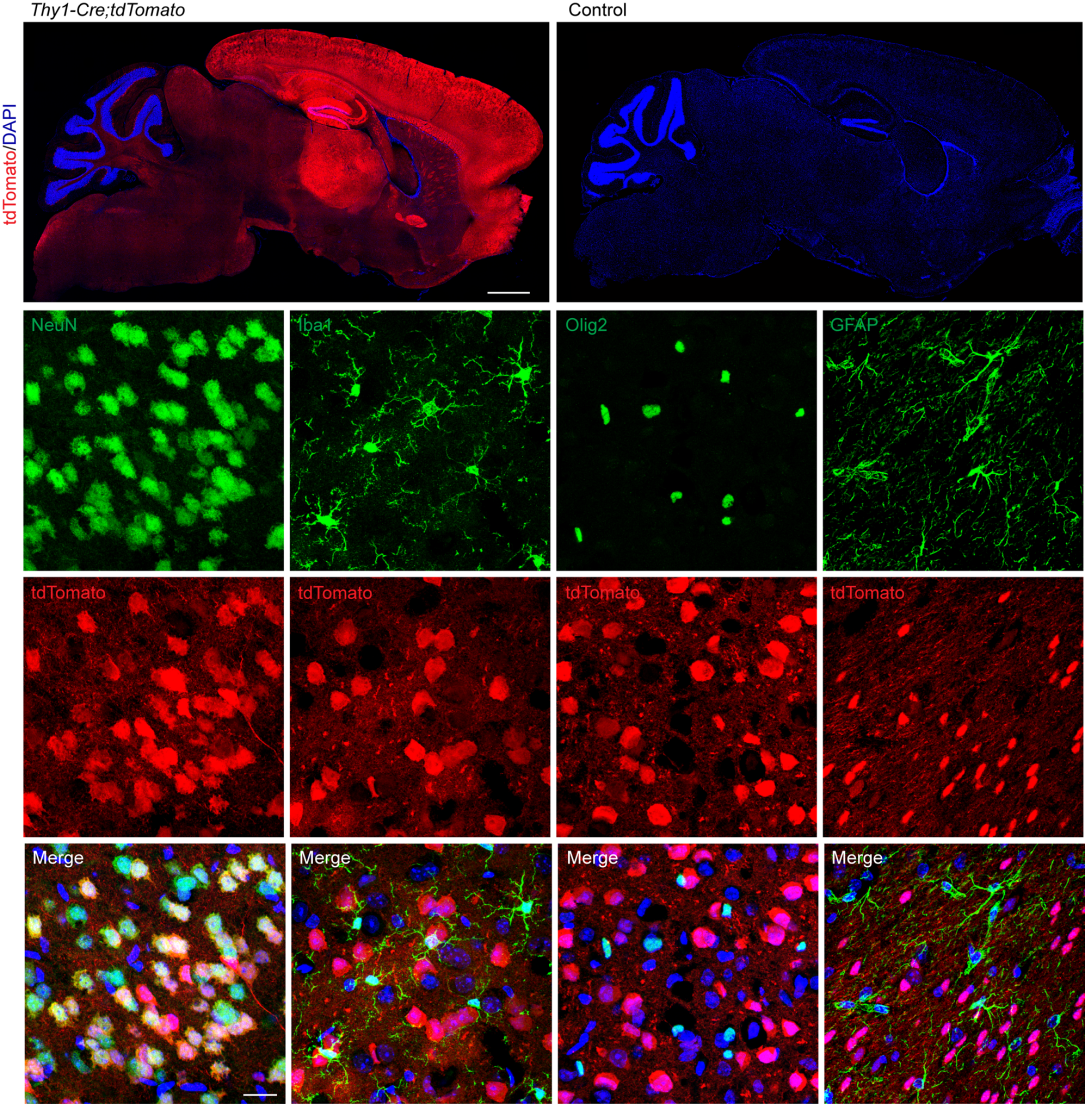
Verification of the cell specificity of the neuron-specific *Gba*-KO mouse. *Thy1-Cre* mice were crossed with tdTomato reporter mice. *Thy1-Cre* drives the specific expression of tdTomato in a subset of neurons (NeuN labeled) but not in microglia (Iba1 labeled), oligodendrocytes (Olig2 labeled), or astrocytes (GFAP labeled). All images (other than tile scans) are from the cortex. Scale bars: 1 mm (tile scan); 20 μm (bottom 3 rows. *n* = 3.

Within *Tmem119-Cre;tdTomato* mice, tdTomato colocalized with Iba1, but not NeuN, Olig2, or GFAP (Figure 2), indicating that *Tmem119-Cre* was expressed in microglia but not neurons, oligodendrocytes, or astrocytes. Within *Thy1-Cre;tdTomato* mice, tdTomato colocalized with NeuN but not Iba1, Olig2, or GFAP (Figure 3). Expression was particularly high in the cortex, hippocampus, and thalamus. In line with previous findings, tdTomato was not expressed in all NeuN^+^ cells (33). This indicated that *Thy1-Cre* is indeed neuron specific but is not expressed in all neurons of the brain. Within *CAG-Cre;tdTomato* mice, the basal levels of tdTomato were elevated throughout the brain. However, high tdTomato expression colocalized strongly with NeuN but not Iba1 or Olig2 (Supplemental Figure 1A; supplemental material available online with this article; https://doi.org/10.1172/jci.insight.179126DS1). Some GFAP^+^ cells exhibited modest tdTomato expression. Pericytes/endothelial cells of the capillaries also appeared to express tdTomato. Profound neuronal expression may reduce the visibility of considerably weaker expression in other cell types like microglia and oligodendrocytes.

To further verify that all cell types in the brains of whole-body *Gba*-KO (*Gba*-wbKO) mice were *Gba* deficient, DNA and protein levels were also assessed. Genomic PCR was used to verify that the *Gba* gene had been appropriately deleted in the cortex, cerebellum, liver, and kidney (Supplemental Figure 1B). Furthermore, GCase was absent from brain tissue, as verified by Western blot (Supplemental Figure 1C). As a result, we concluded that *Gba* was deleted in all cell types of the brain.

As microglia can repopulate (34), we confirmed that the *Gba* deletion in microglia was consistent for up to 12 months. Microglia and other myeloid cells were isolated from *Gba*-mKO mice and control mice at an early- (3–7 months) and late-stage (12 months) time point using CD11b magnetic beads. Genomic PCR verified that the deletion was indeed consistent across both of these time points (Supplemental Figure 2).

We also assessed whether the *Gba* deletion in neuron-specific *Gba*-KO (*Gba*-nKO) mice extended to the peripheral nervous system. Genomic PCR revealed no evidence of a *Gba* deletion in the sciatic nerve, indicating that the peripheral nervous system was at least partially spared (Supplemental Figure 3).

### Ataxia and shortened lifespan occur in Gba-nKO mice but not in Gba-mKO mice.

**Figure 4:**
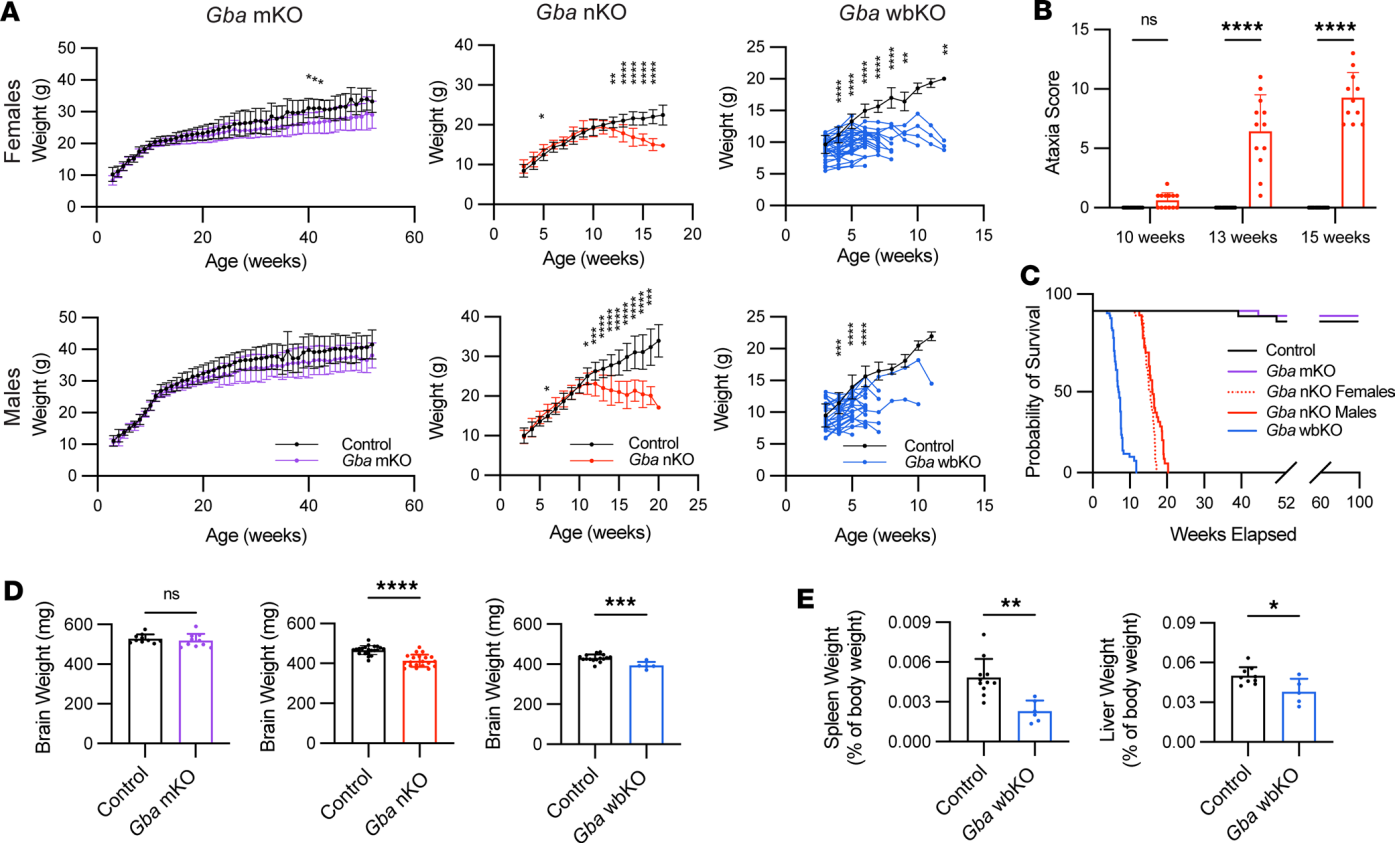
*Gba*-nKO, but not *Gba*-mKO, mice exhibit neurological decline and a shortened lifespan. (**A**) Bodyweight of female and male *Gba*-mKO, *Gba*-nKO, and *Gba*-wbKO mice. Mice were weighed weekly beginning at weaning (when tamoxifen administration began). *Gba*-mKO mouse (*n* = 19–31/group) weights are displayed up to 12 months of age. *Gba*-nKO (*n* = 35–38/group) and *Gba*-wbKO (*n* = 31–57/group) mouse weights are shown up to the end of the lifespan. *Gba*-mKO mice gained weight at a similar rate to that of controls. *Gba*-nKO mice began to decline in weight at about 12 weeks of age. *Gba*-wbKO mice declined in weight rapidly although the initial time point was variable. Mixed-effects model and Holm-Šidák’s multiple comparisons test. (**B**) Ataxia scores based on a battery of tests in *Gba*-nKO mice. Two-way ANOVA and Holm-Šidák’s multiple comparisons test. *n* = 11–13. (**C**) Survival curves of *Gba*-mKO, *Gba*-nKO, and *Gba*-wbKO mice compared with control mice. If survival differences were not significant, results from the different sexes were collapsed. Log-rank (Mantel-Cox) test. *n* = 37–66. (**D**) Brain weight of *Gba*-mKO (*n* = 10), -nKO (*n* = 20–21), and -wbKO (*n* = 5–16) mice compared with that of age-matched controls. Unpaired *t* test. (**E**) Spleen and liver weights of *Gba*-wbKO mice graphed as a percentage of body weight. Unpaired *t* test. *n* = 5–11. *P < 0.05, **P < 0.01, ***P < 0.001, ****P < 0.0001.

Initial observations revealed a stark difference in the phenotype between *Gba*-mKO mice and *Gba*-nKO mice. *Gba*-mKO mice maintained a comparable weight to controls throughout their lifespan (Figure 4A). A battery of tests revealed no ataxia in 12-month-old *Gba*-mKO mice (Supplemental Figure 4). While most *Gba*-mKO mice were collected at 12 months, a subset was monitored up to 15 months (*n* = 3), and no motor deficits or changes in appearance were discernible. In contrast, *Gba*-nKO mice began to decline in weight and developed ataxia by 13 weeks, which progressively worsened over time (Figure 4, A and B). Lifespans of female and male *Gba*-nKO mice were significantly reduced compared with control mice, with median lifespans of 15.6 and 16.1 weeks, respectively (Figure 4C). *Gba*-wbKO mice precipitously declined in weight and reached a humane endpoint soon after (Figure 4, A and C). These mice had a median lifespan of 7 weeks, with no significant difference between sexes. The humane endpoint was determined based on veterinary assessment and substantial weight loss.

By the end of life, *Gba*-nKO and -wbKO mice had reduced brain weights compared with that of age-matched controls (Figure 4D). Additionally, in stark contrast to the hepatosplenomegaly characteristic of GD, *Gba*-wbKO mice exhibited remarkably small spleens and livers (Figure 4E and Supplemental Figure 5A). *Gba*-nKO spleen and liver weights did not differ significantly from those of controls (Supplemental Figure 5, B and C). Based on these results, neuron-specific and ubiquitous deletions of *Gba* cause a rapid phenotypic decline and shortened lifespan, but microglia-specific deletion of *Gba* causes no apparent phenotype for over 1 year of follow-up.

### Sphingolipids characteristic of GD accumulate in Gba-nKO, but not -mKO, mice.

**Figure 5:**
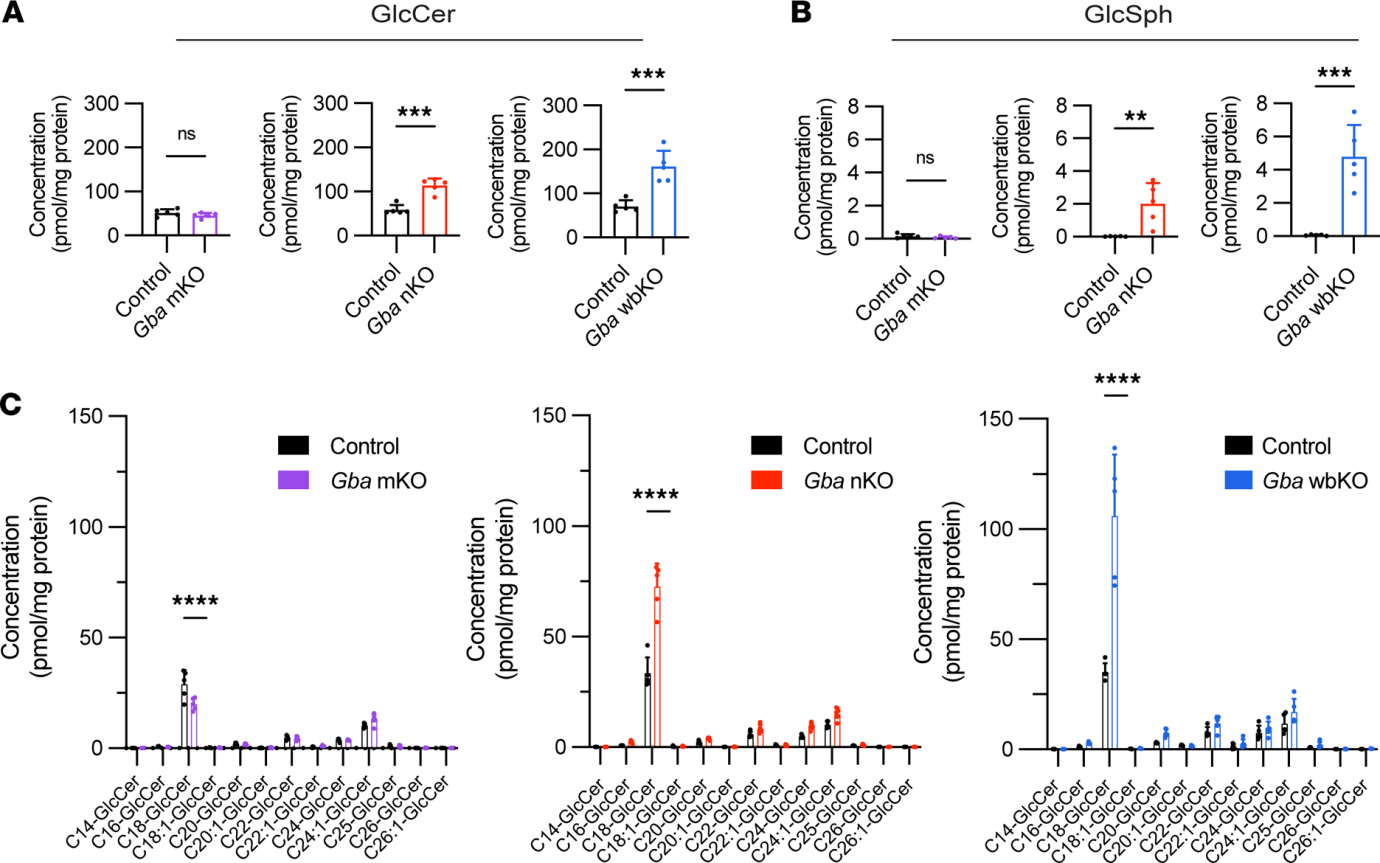
GlcCer and GlcSph levels are elevated in *Gba*-nKO and *Gba*-wbKO mice. *Gba*-mKO, -nKO, and -wbKO brains were collected at 12 months, 16 weeks, and 7–8 weeks, respectively. (**A**) Total GlcCer levels in the brains of *Gba*-mKO, *Gba*-nKO, and *Gba*-wbKO mice. Unpaired *t* test. (**B**) GlcSph levels in the brains of *Gba*-mKO, *Gba*-nKO, and *Gba*-wbKO mice. Unpaired *t* test. (**C**) Individual GlcCer species in the brains of *Gba*-mKO, *Gba*-nKO, and *Gba*-wbKO mice. Two-way ANOVA and Holm-Šidák’s multiple comparisons test. *n* = 5. **P < 0.01, ***P < 0.001, ****P < 0.0001.

GlcCer and GlcSph concentrations were quantified via supercritical fluid chromatography–tandem mass spectrometry (SFC-MS/MS). An accumulation of GlcCer was apparent in both *Gba*-nKO and -wbKO mice but not *Gba*-mKO mice (Figure 5A). In fact, C18-GlcCer was reduced in *Gba*-mKO mice compared with controls but elevated in *Gba*-nKO and *Gba*-wbKO mice (Figure 5C). GlcSph levels were also increased in *Gba*-nKO and -wbKO mice but not in *Gba*-mKO mice (Figure 5B). The standard deviation was also significantly greater in the *Gba*-nKO and *Gba*-wbKO populations compared with control groups.

The sphingolipid that is the primary factor driving toxicity within GD remains uncertain. While GlcCer and GlcSph are potential candidates, some evidence suggests that GlcSph is converted to sphingosine (Sph) via GBA2 and ultimately to sphingosine-1-phosphate (S1P) via Sph kinase, and it is Sph or S1P that is the primary toxic factor in the disease (35). As such, we also examined levels of Cer, Sph, and S1P. *Gba*-mKO mice exhibited slight reductions in C18-Cer and C24:1-Cer levels (Supplemental Figure 6A), but total Cer, Sph, and S1P levels were unchanged (Supplemental Figure 6, D–F). No discernible differences in these lipids were found in *Gba*-nKO mouse brains collected at end of life (16 weeks; Supplemental Figure 6, B and H–J). However, *Gba*-nKO mouse brains collected at 13–14 weeks of age did exhibit elevated S1P levels, an increase in C18:1-Cer levels, and a reduction in C24:1-Cer levels but no change in total Cer or Sph levels (Supplemental Figure 6, C and L–N). DihydroC16-ceramide, dihydrosphingosine, and dihydrosphingosine-1-phosphate levels were also not different from those of controls in *Gba*-mKO and both early- and late-stage *Gba*-nKO mouse brains (Supplemental Figure 6, G, K, and O), indicating no change of sphingolipids in the de novo biosynthesis pathway.

Levels of galactosylceramide (GalCer), a glycosphingolipid enriched in myelin, were slightly elevated in the *Gba*-mKO mice and slightly reduced in the *Gba*-nKO mice (Supplemental Figure 7, A and B). In order to assess how these changes in GalCer may relate to myelination, we determined levels of myelin basic protein (MBP) via Western blot and found a reduction in MBP only in the *Gba*-nKO mice (Supplemental Figure 7, C–H). However, this reduction in MBP did not coincide with changes in Olig2 immunofluorescence, which was not different from controls in the *Gba*-nKO mice (Supplemental Figure 7, I and J). This suggests that the reduction in GalCer and MBP in *Gba*-nKO mice is not due to oligodendrocyte loss but may instead be due to a loss of myelin due to axonal degeneration. Galactosylsphingosine (GalSph) levels were unchanged in *Gba* mKO and *Gba*-nKO mice (data not shown).

### Gba-nKO mice exhibit neurodegeneration not seen in Gba-mKO mice.

**Figure 6:**
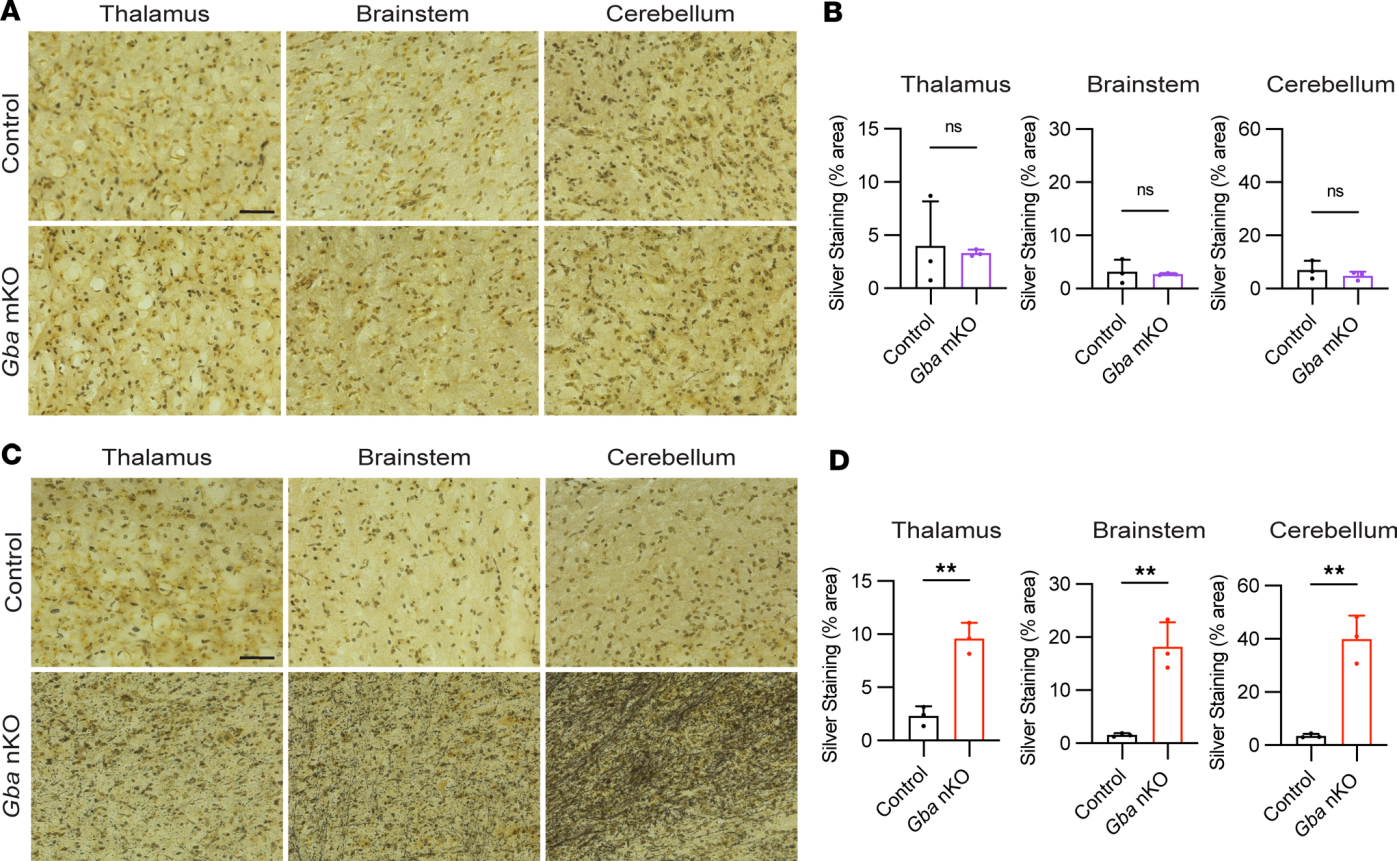
*Gba*-nKO mice exhibit neurodegeneration quantified by silver staining. (**A**) Representative images of thalamus, brain stem, and cerebellum from silver-stained control and *Gba*-mKO mouse brains. (**B**) Quantification of silver staining in *Gba*-mKO brains compared with controls. (**C**) Representative images of thalamus, brain stem, and cerebellum from silver-stained control and *Gba*-nKO mouse brains. (**D**) Quantification of silver staining in *Gba*-nKO brains compared with controls. *Gba*-mKO and -nKO brains were collected at 12 months and 16–18 weeks, respectively. Unpaired *t* test. *n* = 3. Scale bars: 50 μm. **P < 0.01.

To explore whether cell-specific GCase deficiencies were leading to neurodegeneration, a silver stain method was used. Certain components of degenerating neurons, including axons, are argyrophilic (36–40). Thus, silver deposits in the brain following this staining method are indicative of neurodegeneration. While no change was seen in *Gba*-mKO mice (Figure 6, A and B), substantial neurodegeneration was visible in *Gba*-nKO mice in the thalamus, brain stem, and cerebellum (Figure 6, C and D). Quantification revealed significantly more silver deposits in these 3 brain regions in the *Gba*-nKO mice but not in *Gba*-mKO mice. *Gba*-nKO and -mKO groups also exhibited greater variation within groups compared with their control counterparts.

Due to the *Gba* gene’s association with PD, we also investigated whether α-synuclein (aSYN), a prominent protein in PD that can become phosphorylated and aggregates within degenerating dopaminergic neurons (41, 42), may be present in increased quantities. However, total levels of aSYN as well as levels of S129-phosphorylated aSYN appear unchanged in *Gba* nKO and mKO mice compared with controls (Supplemental Figure 8).

### Microgliosis and astrogliosis are present in Gba-nKO brains but not Gba-mKO brains.

**Figure 7:**
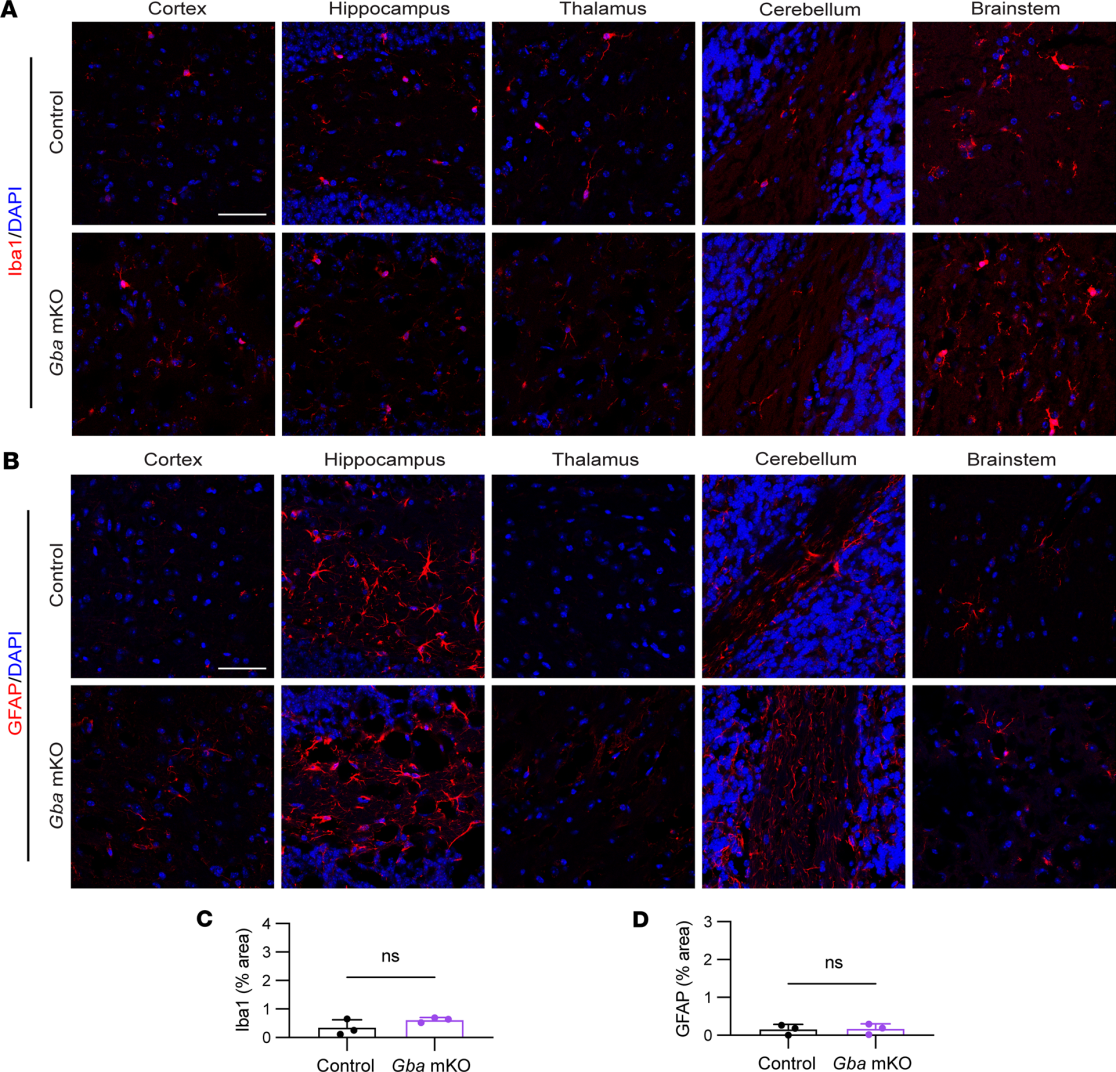
Astrogliosis and microgliosis are not observed within *Gba*-mKO mice following immunofluorescence staining of brain tissue. (**A**) Representative images of immunofluorescence staining of microglia (Iba1) in the cortex, hippocampus, thalamus, cerebellum, and brain stem of *Gba*-mKO mice. (**B**) Representative images of immunofluorescence staining of astrocytes (GFAP) in the cortex, hippocampus, thalamus, cerebellum, and brain stem of *Gba*-mKO mice. (**C** and **D**) Quantification of Iba1 (**C**) and GFAP (**D**) fluorescence averaged across cortex, hippocampus, thalamus, cerebellum, and brain stem in *Gba*-mKO mice compared with age-matched controls. *Gba*-mKO brains were collected at 12 months. Scale bars: 50 μm. Unpaired *t* test. *n* = 3.

**Figure 8:**
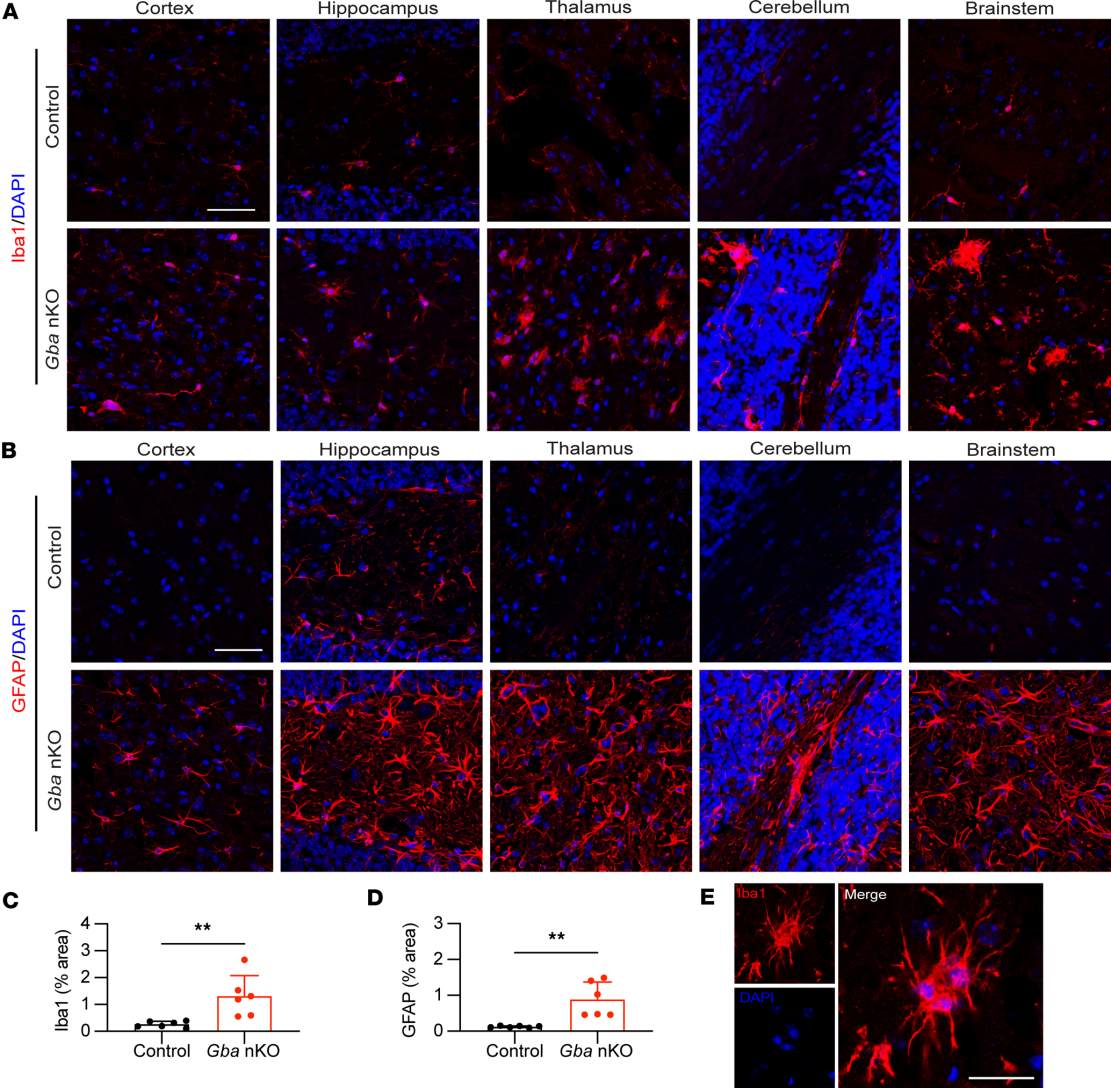
Astrogliosis and microgliosis occur within *Gba*-nKO mice. (**A**) Representative images of immunofluorescence staining of microglia (Iba1) in the cortex, hippocampus, thalamus, cerebellum, and brain stem of *Gba*-nKO mice. (**B**) Representative images of immunofluorescence staining of astrocytes (GFAP) in the cortex, hippocampus, thalamus, cerebellum, and brain stem of *Gba*-nKO mice. (**C** and **D**) Quantification of Iba1 (**C**) and GFAP (**D**) fluorescence in *Gba*-nKO mice compared with age-matched controls. (**E**) An example of a microglial nodule present within *Gba*-nKO mice. *Gba*-nKO brains were collected at 15–18 weeks. Scale bars: 50 μm (**A** and **B**); 25 μm (**E**). Unpaired *t* test. *n* = 6. **P < 0.01.

Immunofluorescence staining was used to investigate whether microglia and astrocytes become activated as a result of cell-specific GCase deficiencies. No notable changes in microglia or astrocytes were found in *Gba*-mKO mouse brains (Figure 7). However, *Gba*-nKO mice exhibited substantial microglial proliferation and changes in morphology indicative of activation, in addition to prominent astrogliosis (Figure 8, A and B). Fluorescence quantification revealed a significant increase in Iba1 and GFAP in *Gba*-nKO brains compared with controls (Figure 8, C and D). This group also exhibited significantly greater variance in fluorescence levels compared with controls. Microglia also appeared to cluster together in *Gba*-nKO brains, forming microglial nodules (Figure 8E).

### Differentially expressed genes indicate a substantial inflammatory response in Gba-nKO mice.

**Figure 9:**
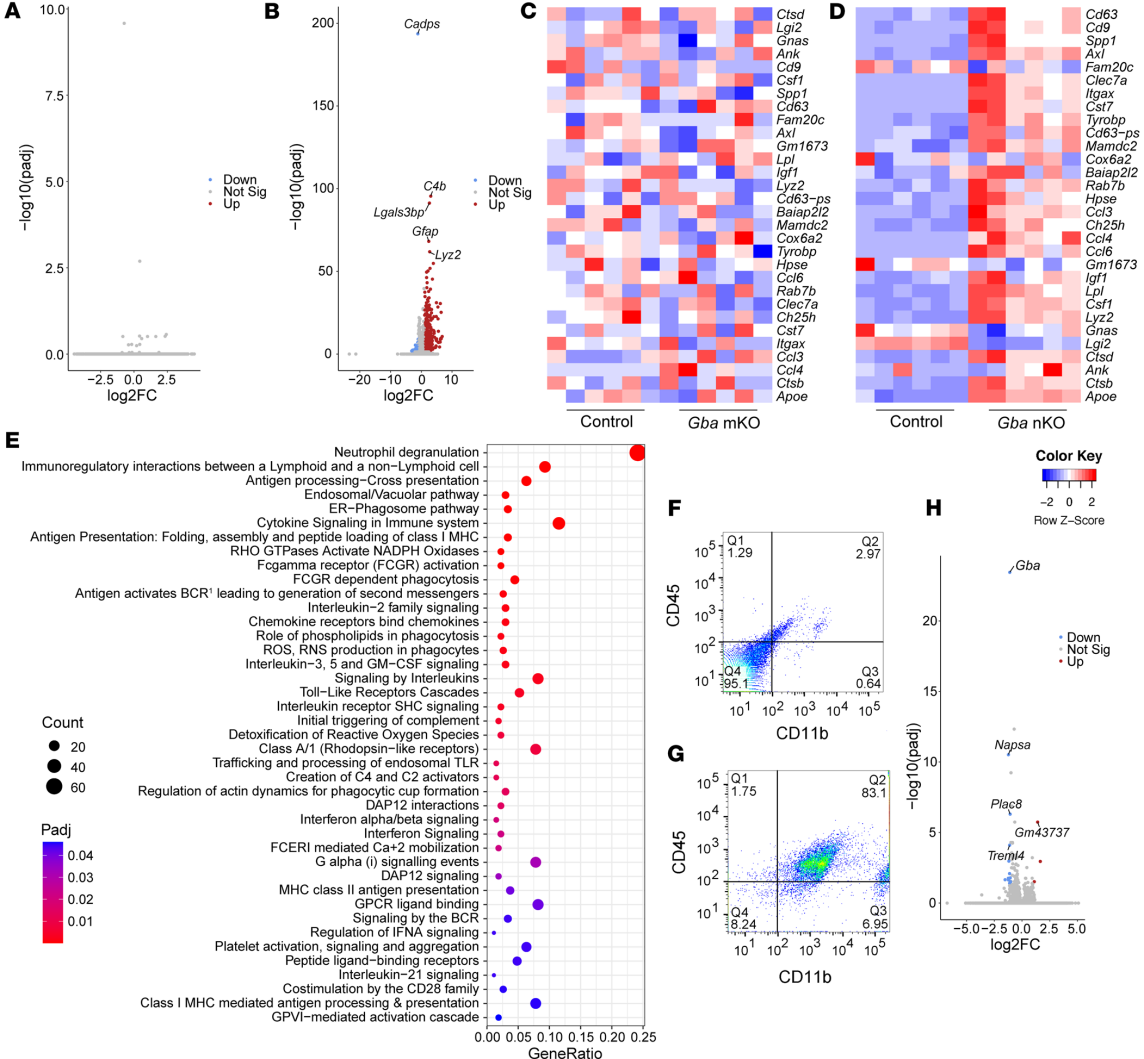
Bulk RNA-Seq of brain tissue reveals substantial changes in gene expression in *Gba*-nKO mice but not in *Gba*-mKO mice. (**A** and **B**) Volcano plots displaying differentially expressed genes (DEGs) in *Gba*-mKO (**A**) and *Gba*-nKO (**B**) mouse brains at 12 months and 15–16 weeks of age, respectively. The 5 most significant DEGs are labeled. *n* = 6. (**C** and **D**) Heatmap of genes related to disease-associated microglia (DAM) in *Gba*-mKO mice (**C**) and *Gba*-nKO mice (**D**) relative to controls. Gene order is computed based on row means. (**E**) Graph of all significantly enriched reactome terms in *Gba*-nKO mice. (**F** and **G**) Cells stained for CD45 and CD11b before (**F**) and after (**G**) microglial isolation. (**H**) Volcano plot displaying DEGs in *Gba*-mKO microglia, isolated from whole brain at 13–15 months of age. The top 5 DEGs are labeled. *n* = 4. |Log2FC| > 1. *P_adj_* < 0.05.

We next explored changes in gene expression by performing bulk RNA-Seq of brain tissue. These data revealed no significantly up- or downregulated genes in the *Gba*-mKO mice relative to age-matched controls (Figure 9A). However, substantial upregulation of genes in the *Gba*-nKO mice was apparent (Figure 9B, Supplemental Table 1, and Supplemental Figure 9A). Of the 10 most significant differentially expressed genes in *Gba*-nKO mice, 5 genes (*Lgals3bp, Lyz2, Cybb, CD68, H2-D1*) have been linked to disease-associated microglia (DAMs) (Supplemental Table 1) (43). Transcriptional changes in these 5 genes were confirmed with qPCR (Supplemental Figure 10). We specifically probed the datasets for genes that were upregulated in DAMs. While these genes showed no apparent changes in *Gba*-mKO mice relative to controls, they showed a clear pattern of upregulation in *Gba*-nKO mice (Figure 9, C and D). Reactome and gene ontology enrichment analysis also suggested a prominent inflammatory response in *Gba*-nKO mice (Figure 9E and Supplemental Figure 9B).

The most significantly upregulated gene was *C4b* (Figure 9B), implicating the complement cascade. “Initial triggering of complement” was also among the significantly enriched reactome terms (Figure 9E and Supplemental Table 2). Interferon and interleukin signaling also appear to play a substantial role in the immune response (Figure 9E and Supplemental Table 2). It has been suggested that pathogen recognition receptors (PRRs) sense sphingolipid accumulation within neurons and then trigger interferon signaling within microglia (44). Based on this hypothesis, we also examined PRR genes within *Gba*-nKO mice (Supplemental Table 3). Of the 17 PRRs that were examined, 11 were previously found to be upregulated in CBE-treated mice and 8 were upregulated in *Gba*-nKO mice (45).

To more closely explore whether any changes in gene expression were occurring within the *Gba*-mKO mice, we isolated microglia (and other CD11b^+^ myeloid cells) from *Gba*-mKO mouse brains using CD11b magnetic beads and performed bulk RNA-Seq of this CD11b^+^ cell population. Unlike before isolation (Figure 9F), the cell population after isolation was predominantly (83.1%) CD45^+^ and CD11b^+^ (Figure 9G), indicating that microglia and other myeloid cells were successfully isolated. Bulk RNA-Seq of this cell population revealed that even among CD11b^+^ cells, hardly any differentially expressed genes were present (Figure 9H). Three genes exhibited increased expression while 13 exhibited decreased expression, the most significant of which was *Gba*, confirming the gene deletion (Supplemental Table 4). Gene ontology enrichment analysis revealed no significant terms associated with this dataset.

## Discussion

The current study explored the cell-type-specific mechanisms associated with neuronopathic GD. While microglial activation and neurodegeneration are associated with GD, the precise role that microglia and neurons play in disease onset is poorly understood. By developing and characterizing cell-type-specific *Gba-*deficient mouse models, we have shown that microglia appear resistant to cell-intrinsic *Gba* deficiencies. *Gba*-mKO mice showed no decline in body weight or lifespan, no development of ataxia or seizures, no prominent sphingolipid accumulation, and no neuroinflammation or neurodegeneration. In sharp contrast, *Gba*-nKO mice did exhibit weight loss, a reduced lifespan, ataxia, sphingolipid accumulation, neurodegeneration, and neuroinflammation, recapitulating the disease state associated with neuronopathic GD. We can, therefore, conclude that a neuron-specific GCase genetic deficiency is sufficient to cause disease while a microglia-specific GCase genetic deficiency is not. While previous GD mouse models have implicated neurons in disease progression (12), to the best of our knowledge, this is the first report of a neuron selective *Gba*-deficient mouse model that does not target other cells in the nervous system. We have shown conclusively that a GCase genetic deficiency in neurons alone is sufficient to cause symptoms of neuronopathic GD.

Microglia are clearly implicated in disease progression. *Gba*-nKO mice demonstrated clear signs of microglial activation, in line with previous neuronopathic GD mouse models (12). Therefore, microglia do appear to play a prominent role in GD disease progression, if not disease onset. Whether microglia-specific GCase deficiencies could worsen the disease state if occurring in tandem with neuron-specific GCase deficiencies remains to be determined.

Our results indicate that cell-intrinsic *Gba* expression is not vital to microglial function. Whether this is because GlcCer catabolism is not an essential microglial function or because microglia are able to compensate for this loss by taking up GCase from surrounding cells is unclear. Previously, in vitro experiments have found that GCase-deficient microglia in culture exhibit changes in morphology and promote inflammation (46). Additionally, lipid storage in microglia has been reported in GCase-deficient mice (29). If *Gba*-mKO microglia can efficiently take up GCase from surrounding cells, this could explain this apparent discrepancy in findings. Neurons are capable of GCase uptake when present in sufficient amounts (23, 47, 48), but whether microglia can perform this process more efficiently must be further explored. An alternative explanation would be that lipid storage only occurs in microglia if the neuronal system is overwhelmed or, alternatively, under conditions of neuroinflammation or other forms of additional stress. Further exploration is needed to understand whether microglial glycosphingolipid catabolism occurs independently or only as a secondary response to neuronal glycosphingolipid catabolism. Boddupalli et al. (30) concluded that a microglia-specific *Gba* deletion results in late-onset neurodegeneration. However, the promoter used in that study, CX_3_Cr1, may be expressed in neurons and macrophages as well as microglia, confounding the interpretation of their results (31, 32). A recent study has directly compared CX_3_Cr1- and Tmem119-inducible Cre lines and found Tmem119 to be more specific, with CX_3_Cr1 also driving expression in macrophages, although Tmem119 did drive expression in a small number of fibroblasts at the blood-brain barrier (49). A *Gba* deletion under the control of the CX_3_Cr1 promoter resulted in GlcCer and GlcSph accumulation at a young age, followed by motor deficits after about 12 months (30). However, we have found that a *Gba* deletion driven by the *Tmem119* promoter results in no GlcCer or GlcSph accumulation and no apparent motor deficits at 12 months of age (Figure 5 and Supplemental Figure 4). This suggests that the changes found in the former group are not due to a microglia-specific deletion. They could instead be the result of a *Gba* deletion within a small population of neurons.

Two recent studies have investigated the mechanisms of GCase deficiencies within glia and neurons of *Droso*p*hila* (50, 51). They have shown that *Gba1b* knockdown in glia damaged neuronal function; caused lysosomal hypertrophy, even in neurons; and led to protein aggregation. In contrast, *Gba1b* knockdown in neurons had no effect. They found that, in *Droso*p*hila*, GlcCer is synthesized in neurons and can be transported to glia for degradation (50). However, this may be unique to *Drosophila*, as our findings show that this is not the primary mechanism for GCase degradation in mice.

Younger *Gba*-nKO mice (13–14 weeks of age) exhibited an accumulation of S1P (fold change [FC] = 1.45) in the brain that was normalized by end of life (16 weeks of age). Previously, it has been suggested that GCase deficiencies lead to a toxic elevation of sphingolipids downstream of GlcCer and GlcSph, which can be ameliorated by downregulating *Gba2* (35). The transient elevation in S1P found in *Gba*-nKO brain tissue could support this theory, although whether this elevation is toxic or protective is not clear. Additionally, GCase can serve as a glucosyltransferase, transferring glucose from GlcCer to cholesterol and producing glucosylcholesterol and vice versa (52, 53). As a result, cholesterol glucosylation activity is reduced in cells derived from patients with GD (53). However, glucosylcholesterol levels are elevated when GBA is inhibited (52). Further research is needed to examine whether an accumulation of primary substrates (GlcCer and GlcSph) or changes in lipid levels further downstream are most detrimental to the system.

The current findings have important implications for the direction of therapeutic developments. The success of ERT targeted to macrophage mannose receptors and the shared lineage between macrophages and microglia may seem to suggest that directing GCase to enzyme-deficient microglia would be beneficial in neuronopathic GD; however, the current findings indicate that it is in fact neurons that must be the primary target for therapeutics. Indeed, neuron-specific targeting of *Gba* gene therapy via AAV has been successful in mice (25).

The *Gba*-nKO mouse has been informative with regards to the extent to which neurons impact GCase deficiencies. It may also be useful in better understanding disease mechanisms that specifically originate within the neuron. Additionally, this mouse model may be useful for future pathogenesis and therapeutic studies.

*Gba*-nKO mice exhibit the same key characteristics seen in other nGD models, namely neurodegeneration, neuroinflammation, and sphingolipid accumulation. The changes in gene expression seen in the *Gba*-nKO mice are also comparable to other GD mouse models. Notably, the top 10 most significant, upregulated genes in both CBE-treated and nestin-Cre mice were also significantly upregulated in *Gba*-nKO mice (44, 54). This transcriptomic profile suggests a profound immune response linked to the complement cascade and cytokine signaling. Previously, complement genes have been found to be elevated in CBE-treated mouse brain tissue (45, 55). Importantly, complement C5a activation has been shown to control GlcCer accumulation and inflammation in GD mice (56). Additionally, reactome and gene ontology enrichment analysis revealed a strong elevation in cytokine signaling. Previous studies have found that interferon signaling genes constitute some of the most upregulated pathways in CBE-treated and nestin-Cre mice, although this interferon signaling has also been shown to be insignificant to the lifespan of these mice (44, 45). Previously described PRRs were also elevated in *Gba*-nKO mice (45).

These tamoxifen-inducible mice enable experimental manipulation concerning the disease time course. In future studies, tamoxifen may be administered at a later time point in order to further explore how aging may interact with GCase deficiencies, a subject of interest within the PD field. While CBE treatment, which chemically inhibits the GBA enzyme, also offers time course flexibility, the tamoxifen system offers several advantages: it does not require continuous daily injection, negates off target effects from CBE, and permits the testing of therapeutics involving active GBA enzyme.

The *Gba* wbKO mouse showed a surprising reduction in spleen and liver weight, contrary to what is typically seen in GD. This discrepancy may be specific to the time point in which this KO was initiated, since previous models initiated before birth, such as the K14-lnl/lnl mice, were not reported to have such an effect (12). While this may limit the relevancy of this whole-body KO, it highlights an interesting developmental effect that should be investigated further in the future, as it may reveal currently unknown mechanisms associated with *Gba* deficiencies.

The present study has introduced a model of neuronopathic GD, which recapitulates known features of the disease, including motor deficits, glycosphingolipid accumulation, neurodegeneration, and neuroinflammation. We have established that a *Gba* deficiency within neurons alone is sufficient to cause disease, whereas a deficiency specific to microglia does not lead to the same outcome — a finding which has important implications for the development of therapeutic strategies.

## Methods

### Sex as a biological variable.

Our study examined male and female animals. Similar findings are reported for both sexes, although some experiments were performed on only 1 sex to reduce variability and sample size.

### Generation of KO mice.

Mice were obtained from The Jackson Laboratory and subsequently bred in-house at the NIH. All mice were housed under the same conditions.

*Gba^fl/fl^*; *Tmem119-CreER^T2^* (microglia-specific *Gba* KO) mice were generated by crossing *Gba^fl/fl^* mice (JAX, 021329) (57) with *Tmem119-CreER^T2^* mice (JAX, 031820) (58). *Gba^fl/+^*; *Tmem119-CreER^T2^* offspring were backcrossed to *Gba^fl/fl^* mice, and *Gba^fl/fl^*; *Tmem119-CreER^T2^* offspring were then crossed to *Gba^fl/fl^* mice. 50% of offspring were then *Gba^fl/fl^*; *Tmem119-CreER^T2^* and 50% were *Gba^fl/fl^* mice, which were used as control littermates (Figure 1B).

*Gba^fl/fl^*; *Thy1-CreER^T2^,-EYFP* (neuron-specific *Gba* KO) mice were generated by crossing *Gba^fl/fl^* mice with *Thy1-CreER^T2^,-EYFP* mice (JAX, 012708) (33, 59). *Gba^fl/+^*; *Thy1-CreER^T2^,-EYFP* offspring were backcrossed to *Gba^fl/fl^* mice, and *Gba^fl/fl^; Thy1-CreER^T2^,-EYFP* offspring were then crossed to *Gbaf^l/fl^* mice. 50% of offspring were then *Gba^fl/fl^; Thy1-CreER^T2^,-EYFP* mice and 50% *Gba^fl/fl^* mice, which were used as control littermates (Figure 1C).

*Gba^fl/fl^*; *CAG-CreER^TM^* (whole-body *Gba* KO) mice were generated by crossing *Gba^fl/fl^* mice with *CAG-CreER^TM^* mice (JAX, 004682) (60). *Gba^fl/+^; CAG-CreER^TM^* offspring were backcrossed to *Gbaf^l/fl^* mice, and *Gba^fl/fl^; CAGG-CreER^TM^* offspring were then crossed to *Gba^fl/fl^* mice. 50% of offspring were then *Gbaf^l/fl^; CAG-CreER^TM^* mice and 50% *Gba^fl/fl^* mice, which were used as control littermates (Figure 1D).

All mice, including floxed *Gba* controls, were fed a tamoxifen citrate diet (500 mg/kg) ad libitum for 5 weeks beginning immediately after weaning (3 weeks). *Gba*-nKO and -wbKO mice were euthanized at the end of their lifespans, which were determined by excessive weight loss and/or veterinary assessment. *Gba*-mKO mice were euthanized at 12 months of age unless otherwise noted.

For validation experiments, *Tmem119-CreER^T2^*, *Thy1-CreER^T2^,-EYFP*, and *CAG-CreER^TM^* mice, were each crossed to *Ai9*(*RCL-tdT)* (JAX, 007909) mice (61). Mice were fed a tamoxifen diet and collected at 6 weeks of age. Throughout the text, these mice are referred to as *Tmem119-Cre;tdTomato*, *Thy1-Cre;tdTomato*, and *CAG-Cre;tdTomato* mice.

### Immunohistochemistry.

Mice were anesthetized with Avertin (250 mg/kg) prior to saline (9.0 g/L NaCl) perfusions. Brains were collected and postfixed with 4% PFA for 24 hours. They were then cryoprotected with 20% sucrose until sinking. 20 μm sagittal sections were cut on a Leica cryostat (CM1950; Leica Biosystems). Slides were briefly washed in PBS before blocking with 5% Normal Goat Serum (Thermo Fisher Scientific); 0.1% Triton X-100 for 1 hour at room temperature. Slides were then incubated with primary antibodies at 4°C overnight. They were subsequently washed with PBS before incubation with secondary antibodies for 1 hour at room temperature. Antibodies are detailed in Supplemental Table 5. Slides were washed once more with PBS and counterstained with DAPI (Thermo Fisher Scientific) before mounting with Prolong Diamond Antifade Mountant (Thermo Fisher Scientific, P36961). Sections were imaged with a Zeiss confocal microscope (Carl Zeiss Microscopy, Model LSM 780). Brightness and channel settings were systematically adjusted across all images. Quantification of *Z*-stacks was performed with Fiji/ImageJ (NIH). One ×40 *Z*-stack per brain region was quantified for each brain section, using 3–6 brain sections per group. The fluorescence quantification of these 4–5 brain regions (brain stem, cerebellum, cortex, thalamus, hippocampus) was then averaged across each brain section.

### Western blot.

Mouse brain and liver were homogenized in RIPA Lysis and Extraction buffer (Thermo Fisher Scientific) with HALT protease inhibitor cocktail (Thermo Fisher Scientific) and HALT phosphatase inhibitor cocktail (Thermo Fisher Scientific). 30 μg of protein per sample was separated on a NuPAGE 4%–12% Bis-Tris gel or a NuPAGE 12% Bis-Tris gel (Thermo Fisher Scientific) under denaturing conditions and transferred to a nitrocellulose membrane with the iBlot2 Blotting System (Thermo Fisher Scientific). Membranes were blocked in 5% nonfat dry milk for 1 hour at room temperature and incubated with primary antibodies at 4°C overnight. Samples were subsequently washed with 5% nonfat dry milk and incubated with secondary antibodies for 1 hour at room temperature. Antibodies are detailed in Supplemental Table 5. Membranes were then developed using the ECL prime Western blotting system (MilliporeSigma, GERPN2232). Western blots were imaged on the Amersham Imager 680 (GE Healthcare Life Sciences) and quantified with Fiji/ImageJ. Relative band intensity was quantified by calculating the FC of all band intensities relative to the first control lane and then by dividing each band’s FC value by their respective actin band’s FC value.

### DNA isolation and PCR.

DNA was isolated from cortex, cerebellum, liver, and kidney with the DNeasy Blood & Tissue Mini Kit (Qiagen) according to the manufacturer’s instructions. DNA was amplified using CloneAmp HiFi PCR Premix (Takara Bio Inc.) and the following *Gba* primers for PCR (5′–3′): GGGTCCTACCACCCATTTCT and GACCTACTTCACTAACAACCGG (Eurofins Genomics LLC). PCR product was then run on an agarose gel.

### Behavioral testing.

A battery of tests was adapted from Guyenet et al. (62) to quantify ataxia. Briefly, mice were tested in 5 categories: hindlimb clasping, stance, ledge walking, gait/tremor, and kyphosis. Each category was scored between 0 and 3, with 0 indicating no abnormality and 3 indicating severe ataxia. The 5 scores were then summed to produce one overall ataxia score for each mouse and time point.

### Mass spectrometry.

Samples were homogenized in RIPA Lysis and Extraction buffer (Thermo Fisher Scientific) with HALT protease inhibitor cocktail (Thermo Fisher Scientific) and HALT phosphatase inhibitor cocktail (Thermo Fisher Scientific). Homogenate was shipped to the Lipidomics Shared Resource at the Medical University of South Carolina where single-phase lipid extraction (acetate/isopropanol/water 60:30:10) and mass spectrometry was performed as previously described (63). Cer, Sph, and S1P were quantified via high-performance liquid chromatography-tandem mass spectrometry (HPLC-MS/MS). GlcCer, GlcSph, and GalCer levels were determined via SFC-MS/MS.

### Neurodegeneration silver staining.

Mice were anesthetized with Avertin (250 mg/kg) prior to saline (9.0 g/L NaCl) perfusion. Brains were collected and postfixed with 4% PFA for 24 hours. They were then cryoprotected with 20% sucrose until sinking. Fresh frozen sections (40 μm) were cut on a cryostat. Silver staining was performed by FD NeuroTechnologies, Inc. using the FD NeuroSilver Kit. Sections were imaged with a Keyence microscope (BZ-X800). Quantification was performed with Fiji/ImageJ, using 3 *Z*-stacks per brain region per slide and 3 slides per sample.

### Microglial isolation and flow cytometry.

Microglial isolation procedures were performed at 4°C. Mice were euthanized with CO_2_, and brains were collected and cut into 8 sagittal sections. Brains were processed with the Adult Brain Dissociation Kit, mouse and rat (Miltenyi Biotec) and magnetically labeled in accordance with the manufacturer’s instructions. Briefly, brains were dissociated under the gentleMACS Octo Dissociator with Heaters (Miltenyi Biotec) and filtered through a 70 μm Smart Strainer (Miltenyi Biotec). Debris and red blood cell removal solutions provided by the manufacturer were applied to the sample. Samples were incubated with CD11b (Microglia) Microbeads, Human and Mouse (Miltenyi Biotec) for 10 minutes prior to magnetic separation on the autoMACS Pro Separator (Miltenyi Biotec).

The percentage of microglia isolated after autoMACS sort was assessed with antibodies directed against CD45 (BD Biosciences, 553080) and CD11b (BD Biosciences, 553311) using the BD FACSAria III flow cytometer (BD Biosciences) and BD FACSDiva Software. Cells that had a fluorescence of more than 2 standard deviations above the unstained control cells were defined as positive.

### RNA extraction, sequencing, and qPCR.

RNA was extracted from 1 hemisphere of whole brain samples with the miRNeasy Mini Kit (Qiagen) in accordance with the manufacturer’s instructions. If isolating from microglia, RNA was extracted using the RNeasy Micro Kit (Qiagen). Samples were submitted to Novogene Inc. for library preparation and bulk RNA-Seq. Briefly, quality of RNA was determined by TapeStation (Agilent Technologies). Library preparation was then performed using the NEBNext Ultra II RNA Library Prep Kit for Illumina (New England BioLabs Inc.). Samples were subsequently sequenced using NovaSeq 6000 PE150 technology (Illumina), generating paired-end reads. High-quality clean reads were mapped to reference genome mm10 using Hisat2 (v2.0.5). FPKM was calculated using featureCounts (v1.5.0-p3). Differential expression was calculated using the DESeq2 R package (1.20.0). Adjusted *P* values were calculated using Benjamini and Hochberg’s approach. Heatmaps and volcano plots were generated using “heatmap.2” and “ggplot” functions in the gplots (3.1.3) and ggplot2 (3.4.2) R Packages. Reactome enrichment analysis was performed through the clusterProfiler R Package. Gene ontology enrichment analysis was performed through The Gene Ontology Resource (https://geneontology.org/). DAM genes were selected from the 30 most significant upregulated DAM genes described by Keren-Shaul et al. (43).

A subset of differentially expressed genes revealed from bulk RNA-Seq was validated with qPCR. RNA was converted to cDNA using Superscript IV VILO Master Mix without DNase (Invitrogen). The following TaqMan Gene Expression Assays (Applied Biosystems) were used with TaqMan Gene Expression Master Mix (Applied Biosystems) for qPCR analysis: Lgals3bp (assay ID: Mm00478303_m1), Lyz2 (assay ID: Mm01612741_m1), Cybb (assay ID: Mm01287743_m1), Cd68 (assay ID: Mm03047343_m1), H2-D1 (assay ID: Mm04208017_mH), and Actb (assay ID: Mm00607939_s1). Gene expression was analyzed using the ΔΔCt method and normalized to actin. “Relative expression” is defined as FC in gene expression relative to control samples.

### Statistics.

Statistical analyses were performed using Prism v9 (GraphPad Software). Log-rank (Mantel-Cox) tests were used to analyze survival curves. Mixed-effects models were used to analyze body weight. Unpaired *t* tests, 2-way ANOVAs, and Holm-Šidák’s multiple comparison tests were used where applicable. All unpaired *t* tests were 2-tailed. Cases in which test assumptions (i.e., equal variance) were not met are stated with test results. Significance for bulk RNA-Seq results was set to *P_adj_* < 0.05 and |Log_2_FC| > 1; otherwise, significance was set to *P* < 0.05. *P* values corresponding to unpaired *t* tests and main effects of 2-way ANOVAs are presented as raw, uncorrected values. *P* values corresponding to Holm-Šidák’s multiple comparison tests and RNA-Seq results have been adjusted (*P_adj_*). All error bars represent standard deviation.

### Study approval.

All mouse experimentation was performed in accordance with and approved by the National Institute of Diabetes and Digestive and Kidney Diseases’ Animal Care and Use Committee.

### Data availability.

RNA-Seq data presented in this report are accessible in NCBI’s Gene Expression Omnibus and can be accessed with the GEO Series accession GSE247436 (64). Values for all data points in graphs are reported in the Supporting Data Values file. All other raw data analyzed and described in this report are available from the corresponding author upon reasonable request.

## Author contributions

HBDD, FMP, and RLP designed the study. HBDD, CB, HZ, YTL, and GT conducted experiments. HBDD analyzed the data and wrote the manuscript. HBDD, CB, HZ, GT, YTL, FMP, and RLP reviewed and edited the manuscript.

## Supplementary Material

Supplemental data

Supporting data values

## Version 1 09/23/2024

In-Press Preview

## Version 2 11/08/2024

Electronic publication
