# Supplementary material for: Deletion of Gba in neurons, but not microglia, causes neurodegeneration in a Gaucher mouse model
Source: JCI Insight. 2024 Nov 8;9(21):e179126. doi: 10.1172/jci.insight.179126 (PMC11601582; doi:10.1172/jci.insight.179126)
Supplement: Supplemental data [file jciinsight-9-179126-s077.pdf]

Deletion of *Gba* in neurons, but not microglia, causes neurodegeneration in a Gaucher mouse model

Duffy et al. (2024)

Supplemental Figures & Tables

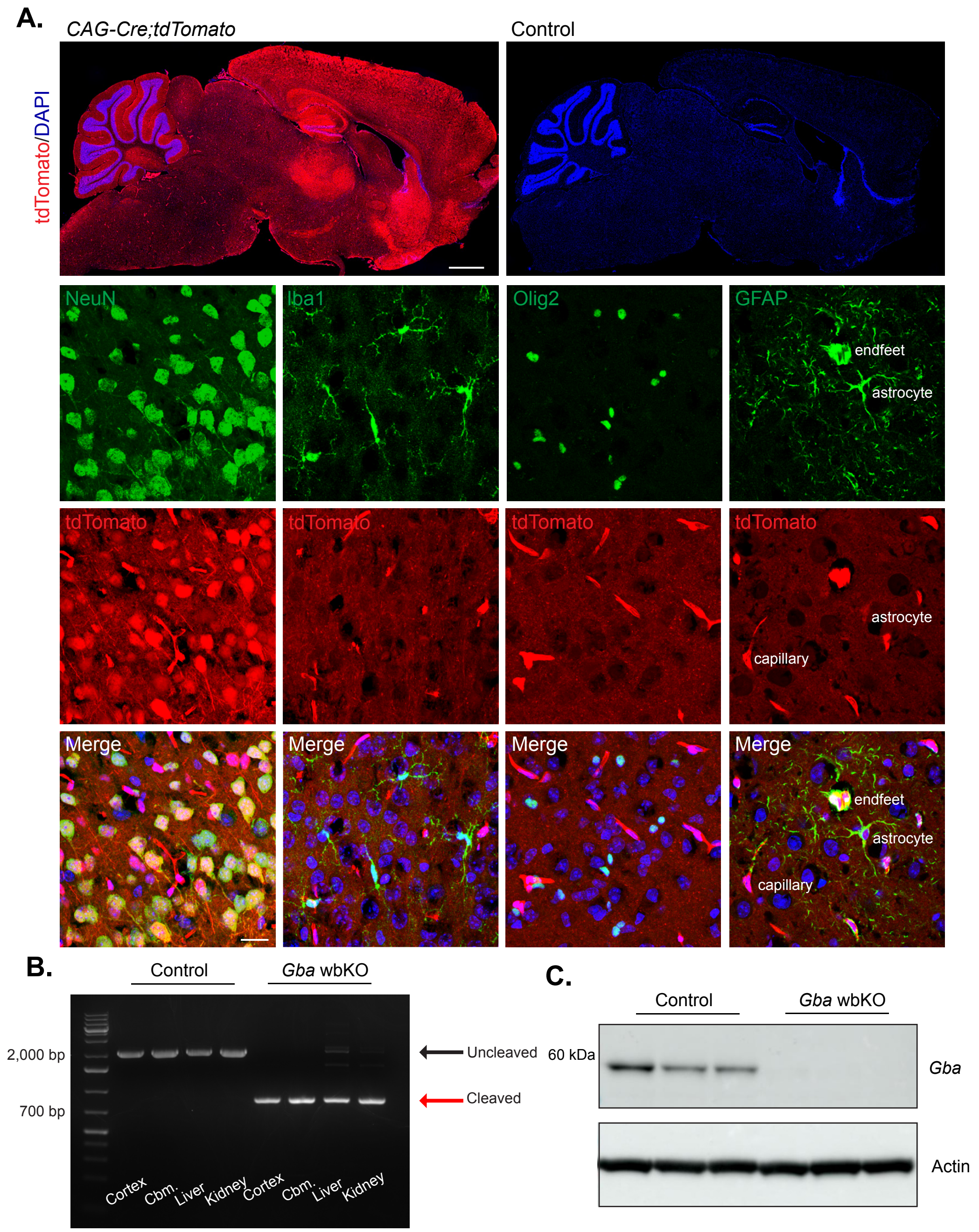

Supplemental Figure 1. Verification of the cell-specificity of the whole-body *Gba* KO mouse. *CAG-Cre* mice were crossed with tdTomato reporter mice. (**A**) *CAG-Cre* drives strong expression of tdTomato in neurons (NeuN labeled), but much weaker expression in microglia (Iba1 labeled) and oligodendrocytes (Olig2 labeled). tdTomato is also highly expressed in pericytes/endothelial cells of the capillaries, which are wrapped by endfeet of astrocytes (GFAP labeled with modest tdTomato expression). All images (other than tile scans) are from the cortex. Scale bars = 1 mm (tile scan) and 20 µm. (**B**) Gel electrophoresis reveals that *CAG-Cre* drives complete cleavage of the floxed *Gba* site in the cortex, cerebellum, liver, and kidney. The cleaved sequence is 841 bp in length while the uncleaved sequence is greater than 2,000 bp. (**C**) Western blot analysis reveals an absence of *Gba* protein driven by *CAG-Cre*. *n* = 3.

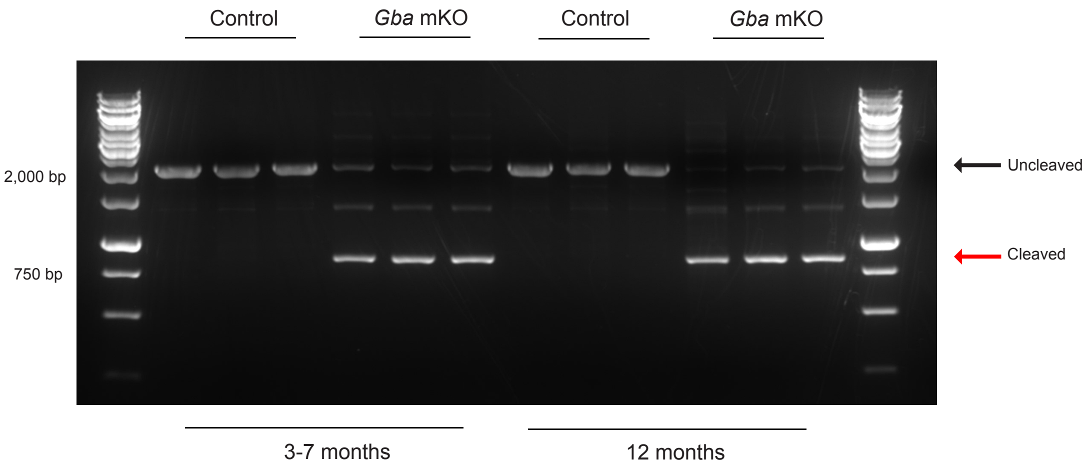

Supplemental Figure 2. Verification of the durability of the microglia-specific *Gba* KO. CD11b$^+$ cells were isolated from brain tissue with magnetic beads for subsequent DNA extraction and PCR. Gel electrophoresis reveals that *Tmem119-Cre* drives cleavage of the floxed *Gba* site in microglia that endures through at least 12 months. The cleaved sequence is 841 bp in length while the uncleaved sequence is greater than 2,000 bp. $n = 3$.

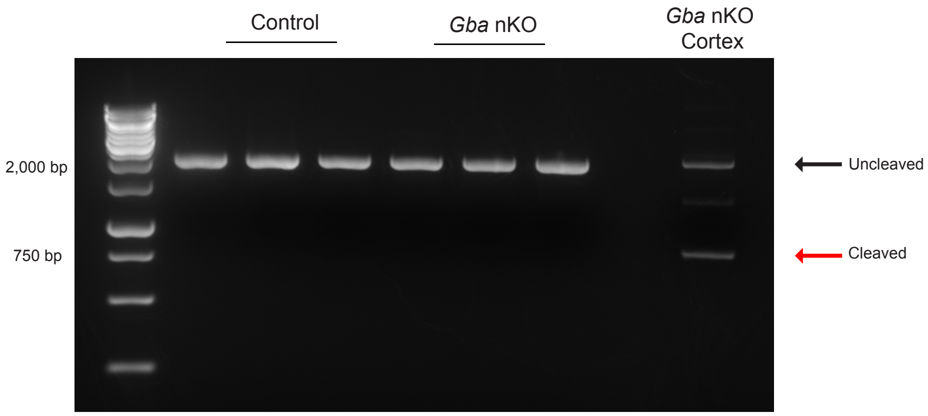

Supplemental Figure 3. Assessment of *Gba* KO within the sciatic nerve. DNA was extracted from the sciatic nerve of control and *Gba* nKO mice at 13-weeks-old. Gel electrophoresis reveals that there is no apparent *Gba* deletion within the sciatic nerve of *Gba* nKO mice, as no band is present at the expected site of the cleaved sequence (841 bp). The uncleaved sequence is expected to be greater than 2,000 bp. DNA from the cortex of *Gba* nKO mice was run alongside sciatic nerve as a positive control (far right lane). This lane shows evidence of cleaved and uncleaved DNA, as would be expected in a neuron-specific deletion. $n = 3$.

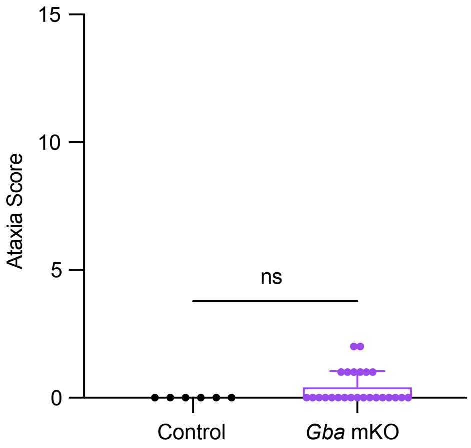

Supplemental Figure 4. *Gba* mKO mice exhibit no motor deficits at 12 months. A battery of ataxia tests revealed no difference between 12-month-old *Gba* mKO and control mice. Unpaired t-test. *n* = 6-25.

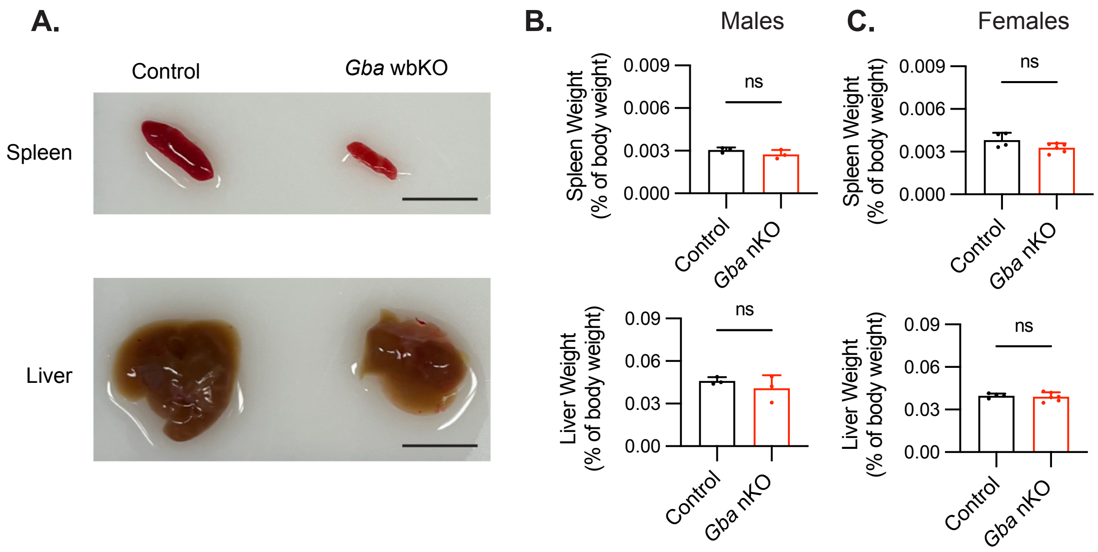

Supplemental Figure 5. *Gba* wbKO, but not *Gba* nKO, mice exhibit reduced spleen and liver sizes. (**A**) Spleen and liver from a control mouse and a *Gba* wbKO mouse collected at the end of the lifespan. Scale bar = 1 cm**.** (**B**) Spleen (p = 0.21) and liver (p = 0.40) weights of 13-week-old male *Gba* nKO mice graphed as a percentage of body weight. n = 3. (**C**) Spleen (p = 0.06) and liver (p = 0.65) weights of 13-week-old female *Gba* nKO mice graphed as a percentage of body weight. Unpaired t-test. n = 4-6.

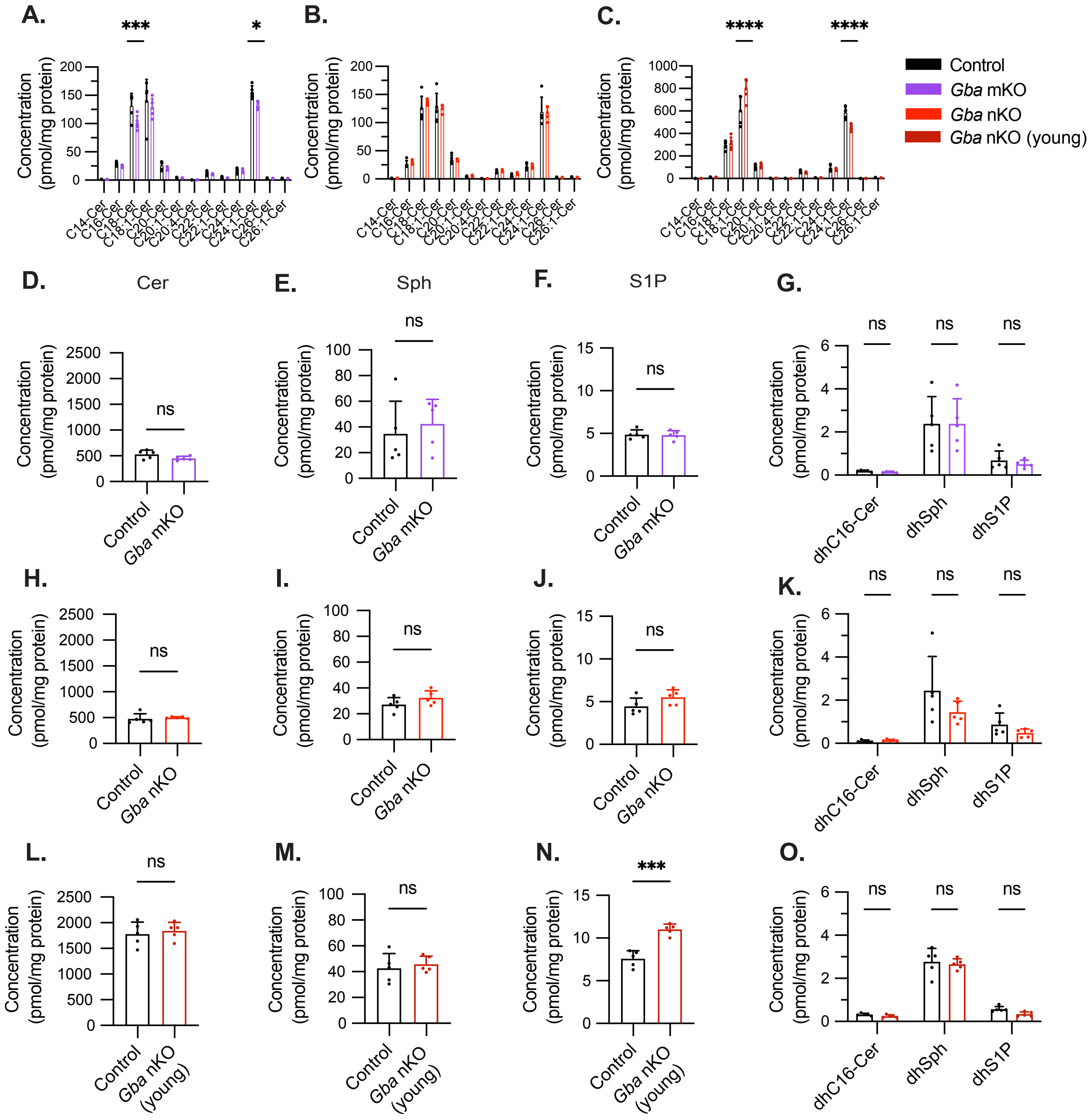

Supplemental Figure 6. *Gba* nKO mice exhibit elevated S1P levels and changes in Cer levels that normalize by the end of the lifespan. Quantified via HPLC-MS/MS. *Gba* mKO brains were collected at 12 months, end-stage *Gba* nKO mice were collected at 16 weeks, and young *Gba* nKO mice were collected at 13-14 weeks. (**A-C**) Levels of Cer species in *Gba* mKO (**A**), end-stage *Gba* nKO (**B**), and younger *Gba* nKO (**C**) mouse brains. (**D-G**) Total Cer (**D**), Sph (**E**), S1P (**F**), and dihydropsphingolipid (**G**) levels in *Gba* mKO mouse brains. (**H-K**) Total Cer (**H**), Sph (**I**), S1P (**J**), and dihydropsphingolipid (**K**) levels in end-stage *Gba* nKO mouse brains. (**L-O**) Total Cer (**L**), Sph (**M**), S1P (**N**), and dihydropsphingolipid (**O**) levels in younger *Gba* nKO mouse brains. Two-way ANOVA, Sidak's multiple comparisons test, and unpaired t-test. $n = 5$. *$p < 0.05$, ***$p < 0.001$, ****$p < 0.0001$.

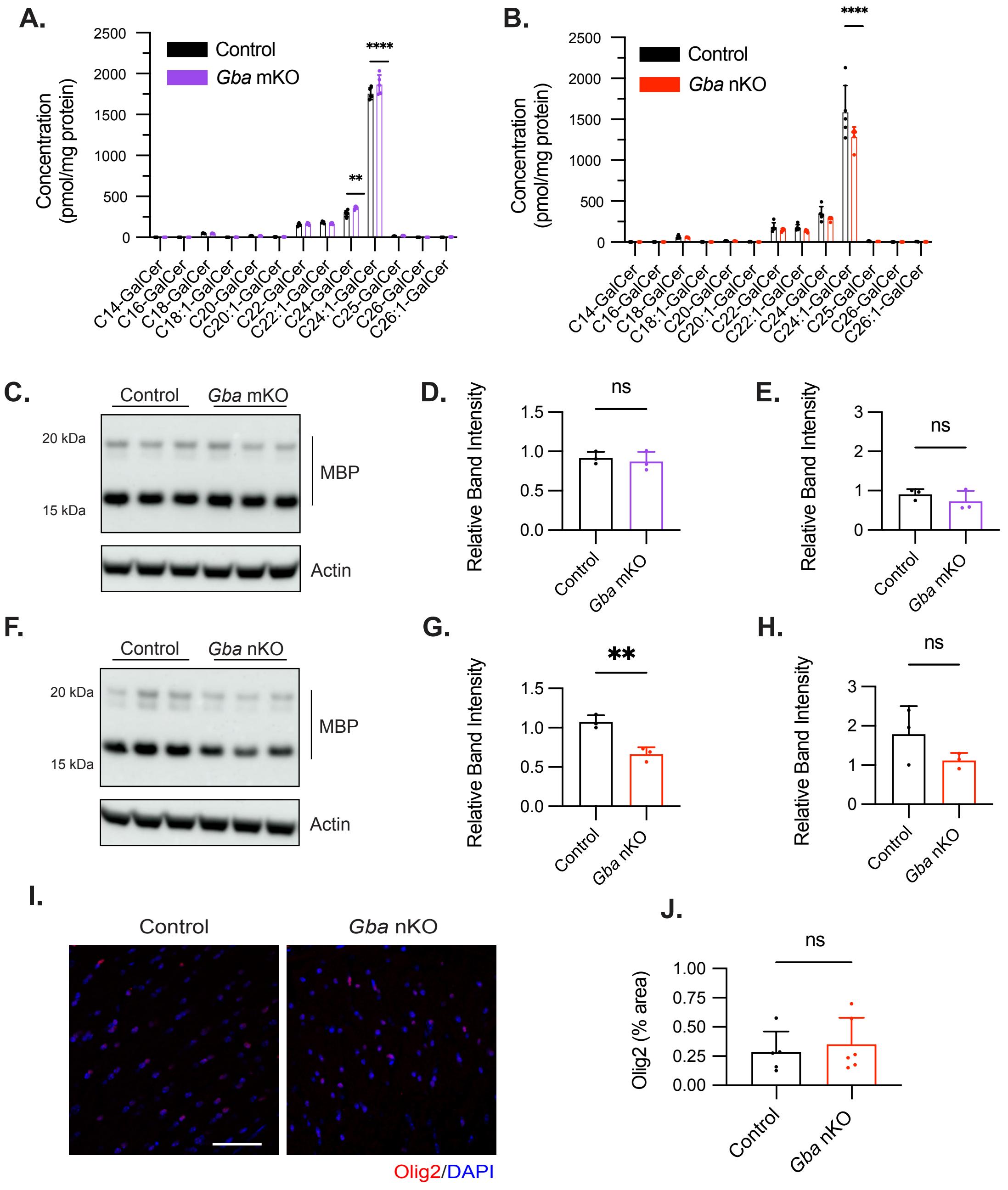

Supplemental Figure 7. *Gba* nKO mice exhibit reductions in GalCer independent of oligodendrocyte levels. (**A-B**) GalCer levels from *Gba* mKO (**A**) and *Gba* nKO (**B**) brains quantified via HPLC-MS/MS. $n = 5$. *Gba* mKO and nKO brains were collected at 12 months and 16 weeks, respectively. (**C-E**) Western blot displaying MBP levels in *Gba* mKO mouse brain (**C**) and quantification of relative band intensity at ~16kDa (**D**) and at ~19-20kDa (**E**). (**F-H**) Western blot displaying MBP levels in *Gba* nKO mouse brain (**F**) and quantification of relative band intensity at ~16kDa (**G**) and at ~19-20kDa (**H**). $n = 3$. *Gba* mKO and nKO brains were collected at 12 months and 15-16 weeks, respectively. (**I**) Representative images of immunofluorescent staining of oligodendrocytes (Olig2) in *Gba* nKO corpus callosum compared to controls. Scale bar = 50 µm. (**J**) Quantification of Olig2 fluorescence in *Gba* nKO mice compared to age-matched controls. $n = 5$-6. *Gba* mKO and nKO brains were collected at 12 months and 15-18 weeks, respectively. Two-way ANOVA, Sidak's multiple comparisons test, and unpaired t-test. $^{**}p < 0.01$, $^{****}p < 0.0001$.

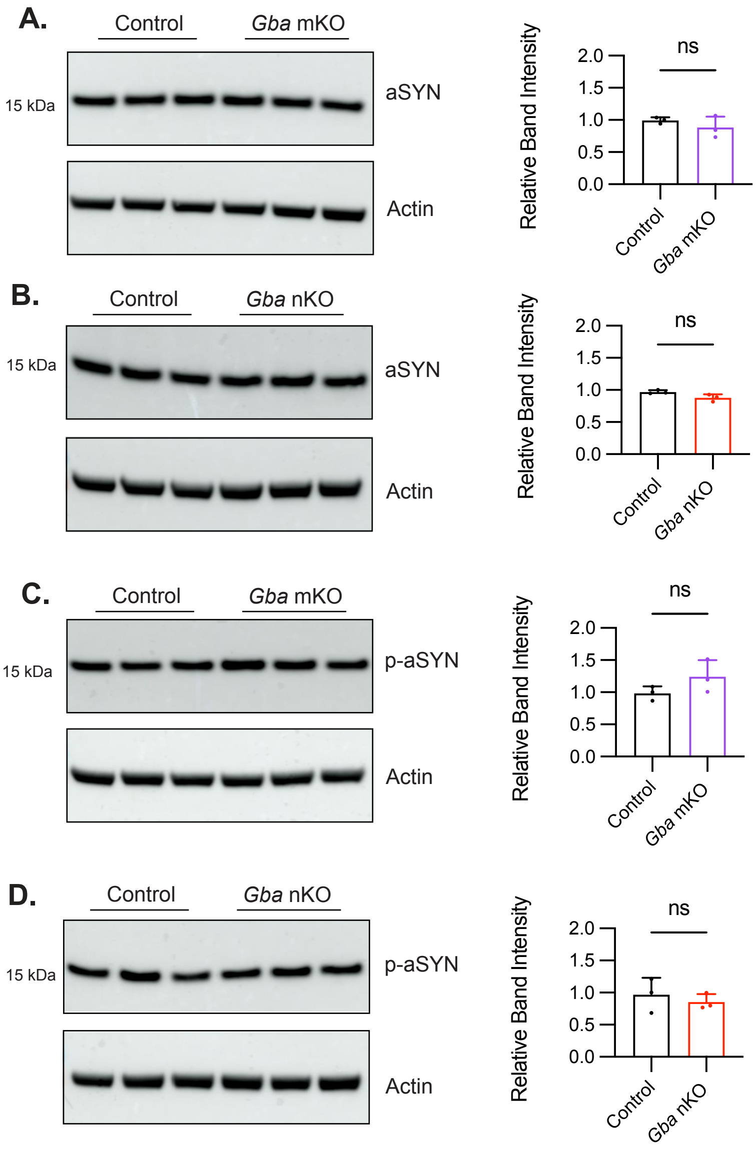

Supplemental Figure 8. Typical aSYN levels are present within *Gba* mKO and *Gba* nKO brains. (**A-B**) Western blots of aSYN in *Gba* mKO (**A**) and *Gba* nKO (**B**) mouse brain (left) and corresponding quantification of relative band intensity (right). (**C-D**) Western blots of phosphorylated aSYN in *Gba* mKO (**C**) and *Gba* nKO (**D**) mouse brain (left) and corresponding quantification of relative band intensity (right). *Gba* mKO and nKO brains were collected at 12 months and 15-16 weeks of age, respectively. Unpaired t-test. *n* = 3.

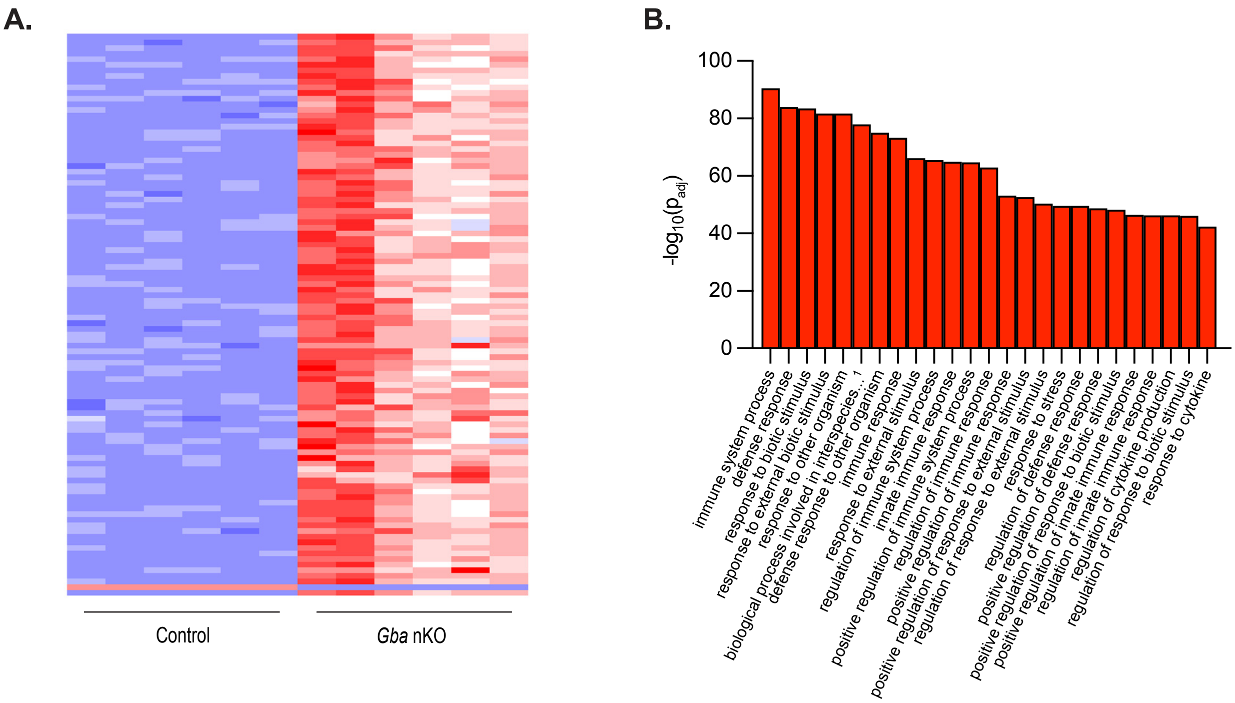

Supplemental Figure 9. Bulk RNAseq from *Gba* nKO mice brain tissue reveals increased gene expression related to a prominent immune response. (**A**) Heat map of the 100 most significant DEGs in *Gba* nKO mice. (**B**) Graph of the top 25 most significant GO terms in *Gba* nKO mice. [1]Biological process involved in interspecies interaction between organisms. $n = 6$. $Padj < 0.05$.

A.

B.

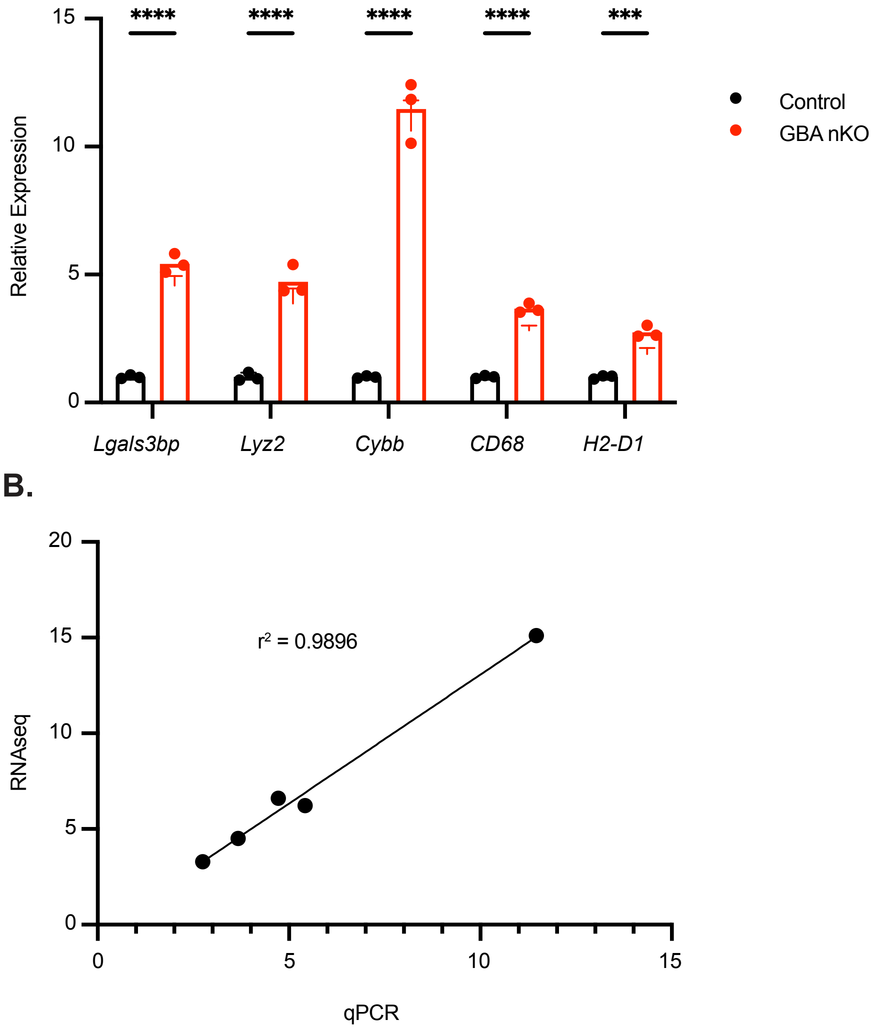

Supplemental Figure 10. qPCR confirms transcriptional changes in DAM genes in GBA nKO mice. (**A**) Changes in gene expression of five DAM related genes, CD68, H2-D1, Lgals3bp, Lyz2, and Cybb, was confirmed using qPCR. Gene expression is graphed as fold change relative to control samples. (**B**) Comparing fold change of these five genes reveals that qPCR and bulk RNAseq are highly correlated. GBA nKO brains were collected at 15-16 weeks of age. Two-way ANOVA, Sidak's multiple comparisons test, and simple linear regression. $n = 3$. ***$p < 0.001$. ****$p < 0.0001$.

Supplemental Table 1. The 100 most significant differentially expressed genes in *Gba* nKO brain tissue relative to controls at 15-16 weeks.

	Gene Name	log_2FC	P_{adj}		Gene Name	log_2FC	P_{adj}
1	*Cadps*	-1.14	2.16E-194	51	*Ifit3b*	2.32	4.49E-24
2	*C4b*	3.07	3.26E-96	52	*AU020206*	2.43	5.56E-24
3	*Lgals3bp*	2.64	5.12E-92	53	*Tyrobp*	2.00	5.74E-24
4	*Gfap*	2.43	7.10E-69	54	*Bst2*	2.55	1.41E-23
5	*Lyz2*	2.73	1.39E-62	55	*Tlr13*	1.97	1.56E-23
6	*Cybb*	3.92	1.57E-55	56	*Lilrb4a*	4.87	2.01E-23
7	*Fcgr2b*	2.84	1.60E-52	57	*Ly9*	4.09	2.53E-23
8	*Cd68*	2.17	5.95E-51	58	*Csf2rb*	2.67	3.66E-23
9	*H2-D1*	1.72	2.90E-50	59	*Usp18*	2.93	5.87E-23
10	*C1qb*	1.72	5.64E-47	60	*Csf3r*	1.84	1.67E-22
11	*Gpnmb*	3.62	5.10E-46	61	*Ctsh*	1.52	3.98E-22
12	*C1qc*	1.88	7.29E-44	62	*Ifi207*	3.61	3.98E-22
13	*A2m*	1.79	7.20E-41	63	*Lcn2*	5.45	5.28E-22
14	*Serpina3n*	2.97	5.71E-40	64	*Siglec1*	3.33	6.34E-22
15	*Cd84*	2.39	1.42E-38	65	*Pld4*	1.53	1.56E-21
16	*Grn*	1.15	4.03E-38	66	*Ddx60*	3.15	3.47E-21
17	*Vim*	1.20	1.76E-35	67	*Cyba*	1.74	5.36E-21
18	*C3ar1*	2.52	1.02E-34	68	*Trem2*	2.31	6.35E-21
19	*Ptprc*	2.29	1.84E-34	69	*Ctsz*	1.33	1.72E-20
20	*Ctss*	2.04	1.30E-33	70	*B2m*	1.58	2.16E-20
21	*H2-Q4*	2.18	1.39E-32	71	*Aspg*	1.63	2.22E-20
22	*Ifi204*	4.92	8.77E-32	72	*Ifih1*	1.38	7.17E-20
23	*Cybrd1*	1.68	1.06E-31	73	*Psmb8*	2.17	1.47E-19
24	*Itgax*	6.19	2.90E-31	74	*Cd53*	1.49	2.05E-19
25	*Slc11a1*	2.59	2.21E-30	75	*Tent5c*	1.76	4.07E-19
26	*Irgm1*	1.69	3.73E-30	76	*Stat1*	1.38	5.08E-19
27	*Nckap1l*	1.47	7.50E-30	77	*Lag3*	2.53	5.24E-19
28	*Clec7a*	5.41	3.64E-29	78	*Ifit1*	2.81	2.22E-18
29	*Rtp4*	2.46	5.09E-29	79	*Nlrc5*	2.31	2.23E-18
30	*Plek*	1.68	2.60E-28	80	*Ifi27*	1.01	2.46E-18
31	*H2-Q6*	2.77	3.18E-28	81	*H2-K1*	1.62	3.30E-18
32	*Mpeg1*	2.28	4.20E-28	82	*Lpl*	1.27	5.99E-18
33	*Tlr2*	1.93	4.34E-28	83	*Flnc*	1.64	1.26E-17
34	*Tap1*	1.89	5.49E-28	84	*Myo1f*	2.27	4.31E-17
35	*Inpp5d*	1.09	1.12E-27	85	*Ptpn6*	2.10	7.63E-17
36	*Oasl2*	3.34	1.17E-27	86	*Slamf9*	3.10	7.98E-17
37	*Slc15a3*	2.85	1.50E-27	87	*Trim30a*	2.08	8.22E-17
38	*Itgb2*	2.15	2.57E-27	88	*Capg*	2.06	1.77E-16
39	*Abca1*	1.17	2.64E-27	89	*H2-Q7*	3.00	1.88E-16
40	*Parp14*	1.83	2.88E-27	90	*Cd33*	1.12	2.06E-16
41	*Irf8*	1.86	8.27E-27	91	*Arhgap45*	1.34	2.52E-16
42	*Cxcl10*	6.46	5.83E-26	92	*Ifit2*	1.15	2.99E-16
43	*Lgals3*	3.31	1.23E-25	93	*Mx1*	3.56	2.80E-15
44	*Fcgr3*	1.74	4.18E-25	94	*Apobec1*	1.99	2.87E-15
45	*Laptm5*	1.36	4.54E-25	95	*Trim25*	1.16	3.13E-15
46	*Fcer1g*	1.86	4.84E-25	96	*H2-T23*	1.07	3.63E-15
47	*Eif2ak2*	1.33	1.21E-24	97	*Cd14*	1.91	3.63E-15
48	*Ctsd*	1.11	3.06E-24	98	*Fermt3*	1.38	5.05E-15
49	*Cd52*	2.86	3.69E-24	99	*Cyth4*	1.33	5.34E-15
50	*C1qa*	1.94	4.49E-24	100	*Ccl3*	4.02	6.25E-15

Supplemental Table 2. All significant differentially expressed genes within 3 significant reactome pathways in *Gba* nKO brain tissue relative to controls at 15-16 weeks.

Initial Triggering of Complement (R-MMU-166663)

Gene Name	log_2FC	P_{adj}
C4b	3.07	3.26E-96
C1qb	1.72	5.64E-47
C1qc	1.88	7.29E-44
C3ar1	2.52	1.02E-34
C1qa	1.94	4.49E-24
Colec11	2.05	2.82E-03

Interferon Signaling (R-MMU-913531)

Gene Name	log_2FC	P_{adj}
Eif2ak2	1.33	1.21E-24
Usp18	2.93	5.87E-23
Ptpn6	2.10	7.63E-17
Trim25	1.16	3.13E-15
Ddx58	1.49	1.01E-14
Irf9	1.02	1.06E-12
Mx2	2.02	2.73E-12
Oasl1	3.71	1.45E-11
Isg15	2.59	2.10E-08
Uba7	1.53	3.75E-08
Ube2l6	1.25	8.17E-08
Hspa1a	-1.22	2.81E-03
Hspa1b	-1.16	2.17E-02

Signaling by Interleukins (R-MMU-449147)

Gene Name	log_2FC	P_{adj}
Inpp5d	1.09	1.12E-27
Csf2rb	2.67	3.66E-23
Usp18	2.93	5.87E-23
Psmb8	2.17	1.47E-19
Nlrc5	2.31	2.23E-18
Ptpn6	2.10	7.63E-17
Osmr	1.41	3.37E-11
Vav1	1.35	3.08E-08
Il10ra	1.11	3.37E-07
Gsdmd	1.32	1.09E-06
Il20rb	1.00	2.77E-06
Casp1	1.04	4.51E-04
Hck	1.14	5.99E-04
Il2rb	1.93	1.25E-03
Il21r	1.27	1.26E-03
Il1a	1.71	1.94E-03
Il1rl2	1.44	3.64E-03
Ager	1.65	2.26E-02
Il2rg	1.29	3.21E-02
Ebi3	1.37	3.23E-02

Supplemental Table 3. Pathogen recognition receptor genes in *Gba* nKO brain tissue relative to controls at 15-16 weeks.

**Pathogen Recognition Receptors**			
**Gene Name**	**log_2FC**	**P_{adj}**	**Significant?[A]**
Tlr1	2.85	4.20E-11	Yes[B]
Tlr2	1.93	4.34E-28	Yes[B]
Tlr3	0.46	7.75E-03	No[B]
Tlr4	0.51	0.162	No[B]
Tlr5	0.25	0.825	No
Tlr6	1.15	0.133	No
Tlr7	1.37	1.54E-08	Yes[B]
Tlr8	0.97	1.000	No
Tlr9	1.03	1.51E-04	Yes
Tlr11	0.48	1.000	No
Tlr12	1.83	1.81E-04	Yes
Tlr13	1.97	1.56E-23	Yes[B]
Ddx58	1.49	1.01E-14	Yes[B]
Ifih1	1.38	7.17E-20	Yes[B]
Nod2	0.82	0.069	No[B]
Mb21d1	0.70	0.078	No[B]
Tmem173	0.80	1.27E-02	No[B]

[A]$|log2FC| > 1$, $P_{adj} < 0.05$.

[B]Significantly increased in CBE-treated mice (45).

Supplemental Table 4. Significant differentially expressed genes in *Gba* mKO microglia relative to controls at 13-15 months.

Gene Name	log_2FC	P_{adj}
Gba	-1.11	3.60E-24
Napsa	-1.23	3.04E-11
Plac8	-1.07	4.95E-07
Gm43737	1.41	1.81E-06
Treml4	-1.12	7.92E-05
Rspo1	-1.18	1.07E-03
Prlr	1.65	1.11E-03
S1pr5	-1.12	8.10E-03
Sirpb1c	-1.07	1.64E-02
Cd300e	-1.23	1.73E-02
Fabp4	-1.52	2.21E-02
Acp5	-1.22	2.29E-02
Dusp5	-1.05	3.02E-02
Htr2c	1.11	3.04E-02
Grap2	-1.12	3.93E-02
Rasgrp1	-1.03	4.07E-02

Supplemental Table 5. Antibodies used for western blotting and immunohistochemistry.

Antibody	Source	Catalog #	Dilution	Purpose	Company
Anti- α-synuclein	Rabbit Polyclonal	2642	1:10,000	Western	Cell Signaling Technology
Anti-Β-actin	Mouse Monoclonal	Ab49900	1:20,000	Western	Abcam
Anti-GC	Rabbit Polyclonal	88162	1:1,000	Western	Cell Signaling Technology
Anti-GFAP	Rabbit Polyclonal	Ab7260	1:500	IHC	Abcam
Anti-Iba1	Rabbit Polyclonal	019-19741	1:500	IHC	Wako
Anti-MBP	Rabbit Polyclonal	PA5-78397	1:10,000	Western	Thermo Fisher Scientific
Anti-Olig2	Rabbit Monoclonal	Ab109186	1:500	IHC	Abcam
Anti-phospho-α-synuclein	Rabbit Monoclonal	23706	1:10,000	Western	Cell Signaling Technology
Dylight Alexa Fluor^{TM} 594	Goat Anti-Rabbit	35561	1:500	IHC	Thermo Fisher Scientific
Peroxidase Conjugated	Goat Anti-Rabbit	AP132P	1:3,000	Western	Millipore Sigma
Alexa-647 Conjugated	Goat Anti-Rabbit	A32733	1:400	IHC	Thermo Fisher Scientific
